# Encephalitozoonosis in Pet Rabbits: Epidemiology, Pathogenesis, Immunology and Consensus on Clinical Management

**DOI:** 10.3390/ani16020346

**Published:** 2026-01-22

**Authors:** Emma Keeble, Frank Kϋnzel, Fabiano Montiani-Ferreira, Jennifer Graham, Edita Jeklová, Sari Kanfer, Angela Lennox, Guillaume Desoubeaux, Ethan Biswell, Carolyn Cray, Anja Joachim

**Affiliations:** 1 The Royal (Dick) School for Veterinary Studies, The Roslin Institute, The University of Edinburgh, Roslin EH25 9RG, UK; emma.keeble@ed.ac.uk; 2 Clinic for Small Animals, Clinical Department for Small Animals and Horses, University of Veterinary Medicine, 1210 Vienna, Austria; frank.kuenzel@vetmeduni.ac.at; 3 Departamento de Medicina Veterinaria, Universidade Federal do Paranã, Curitiba 80035-050, PR, Brazil; montiani@ufpr.br; 4 Department of Pathology & Laboratory Medicine, University of Miami Miller School of Medicine, Miami, FL 33136, USA; 5 Graham Veterinary Consulting, LLC, Madison, AL 35758, USA; 6 Veterinary Research Institute, 621 00 Brno, Czech Republic; edita.jeklova@vri.cz; 7 Exotic Animal Veterinary Center, Pasadena, CA 91107, USA; bunnyvet@eavcpasadena.com; 8 Avian & Exotic Animal Clinic, Indianapolis, IN 46268, USA; alennox@exoticvetclinic.com; 9 Research Center for Respiratory Diseases, Universite de Tours, 37044 Tours, France; guillaume.desoubeaux@univ-tours.fr; 10 Unit of Parasitology-Mycology-Tropical Medicine, Hospital Bretonneau, 37032 Tours, France; 11 Biosphere Lab, Louisville, KY 40299, USA; 12 Institute of Parasitology, Department of Biological Sciences and Pathobiology, University of Veterinary Medicine, 1210 Vienna, Austria; anja.joachim@vetmeduni.ac.at

**Keywords:** *Encephalitozoon*, lagomorph, microsporidia, *Oryctolagus cuniculus*

## Abstract

Members of the microsporidial genus *Encephalitozoon* have the capacity to infect both mammals and birds, and *E. cuniculi* is most commonly found in rabbits. With a seroprevalence ranging up to 85%, *E. cuniculi* can be a problem in pet rabbits as well as in food production and laboratory animal science. While most infections are likely subclinical, there are three main clinical presentations: neurological, renal, and ocular. Typical clinical signs including vestibular disease and phacoclastic uveitis may develop with initial or relapsing infection, while renal infection is usually progressive and associated with non-specific clinical signs. High-sensitivity/specificity ante mortem diagnostic options are lacking, and serological testing most often provides adjunct rather than definitive information such that physical examination and other diagnostics are used more so for ruling out other differentials and comorbidities, rather than confirming infection. In the veterinary community, treatment regimens are variable given the lack of thorough studies and a consensus. The aim of this document is to present the available literature to give a concise review of this organism and its infection of rabbits as well as to propose guidelines and protocols for diagnostics and treatment regimens. In addition, the current challenges and recommendations for further studies are discussed.

## Simple Summary

*Encephalitozoon cuniculi* infection in rabbits is challenging due to the high seroprevalence, treatment regimens based on older literature, and the paucity of active research. Given the lack of agreement in the veterinary community and modern robust studies, there is both high variance in the base knowledge of this agent and the disease processes as well as considerable variability in the application of therapies. The aim of this review is to provide a focused presentation regarding the biology and clinical presentations with a consensus on the use of diagnostics and treatment regimens.

## 1. Introduction

Microsporidia are eukaryotic single-cell pathogens with the capacity of spore-forming inside the intracellular compartment of their host. Recently, they have been described to be related to the phylum of fungi [1,2]. While they have been long overlooked, microsporidia have now been classified by the National Institutes of Allergy and Infectious Diseases and the Centers for Disease Control and Prevention as Category B biodefense priority pathogens [3].

The life cycle of microsporidia is initiated by infection of target cells as the polar tube is extruded from the spore and inserted into the host cell. The parasite sporoplasm enters the host cell to form a new generation of parasites by asexual multiplication, which is completed by the formation of spores that are released from the cell to either be excreted or to infect additional host cells in the same organism [4]. The primary mode of transmission is spores shed in excreta that can be disseminated in the environment and remain viable for weeks to months, and, due to their small size, can prevail in water bodies, which means water-borne infections are highly prevalent. For mammals, vertical infection from the dam to the fetus can occur [4,5]. In addition, tick-borne transmission has recently been proposed [6].

The phylum Microsporidia comprises a wide range of genera and >1000 species that can infect invertebrates and vertebrates [7]. A global meta-analysis revealed high seroprevalence for microsporidia in mammalian hosts, and DNA of 7 species and >700 genotypes were described from 92 countries, with high rates in Northern Europe and South Africa [8]. However, global prevalence and specific geographical risk factors are not easy to define given the ubiquitous nature, large and phylogenetically diverse host spectrum, and genetic diversity of the microsporidia in general. In domestic mammals, the genus *Encephalitozoon* is the most common and represented mostly by two species, *E. cuniculi* and *E. intestinalis,* whereas *E. hellem* primarily infects birds [9]. Humans, especially those who are immunocompromised, such as HIV-infected patients and solid-organ transplant recipients [1,10], as well as travelers and children in countries with poor hygiene [11,12], can be affected by different zoonotic *Encephalitozoon* species as well as *Enterocytozoon bieneusi*, a species where cattle, pigs, and rodents are considered as the reservoir for zoonotic transmission [13,14,15]. Within the different microsporidian species, genotypes with varying host preferences are described [9]. For example, *E. cuniculi* is currently subdivided into four different genotypes (Ec I to IV) according to the number of repeat sequences in their ribosomal RNA internal transcribed spacer regions [16]. Genotype Ec I infects primarily rabbits and humans; Ec II mice, rats, cats, arctic foxes, and humans; Ec III is described in dogs in the USA and South Africa, South American monkeys, steppe lemmings, and humans (and may comprise several genotypes); and Ec IV has been detected in humans, dogs, and cats [17,18].

This review of recent and major publications on rabbit encephalitozoonosis and all other topics discussed herein were retrieved from scientific databases including PubMed, Scopus, and Google Scholar. We present suggestions for the management of *E. cuniculi* infection in rabbits in its different forms, including diagnosis, treatment, and prevention, with a focus on peer-reviewed experimental and clinical studies in conjunction with previously unpublished patient data provided by experts in the field of exotic companion mammal medicine. The latter is indicated throughout all sections as personal communications. This work is the result of discussions by an expert panel as part of the first ECUN 360: A Virtual Conference on *E. cuniculi* Infection of Rabbits held on 5 October 2024.

## 2. Epidemiology and Pathogenesis of *E. cuniculi* in Rabbits

**Figure 1:**
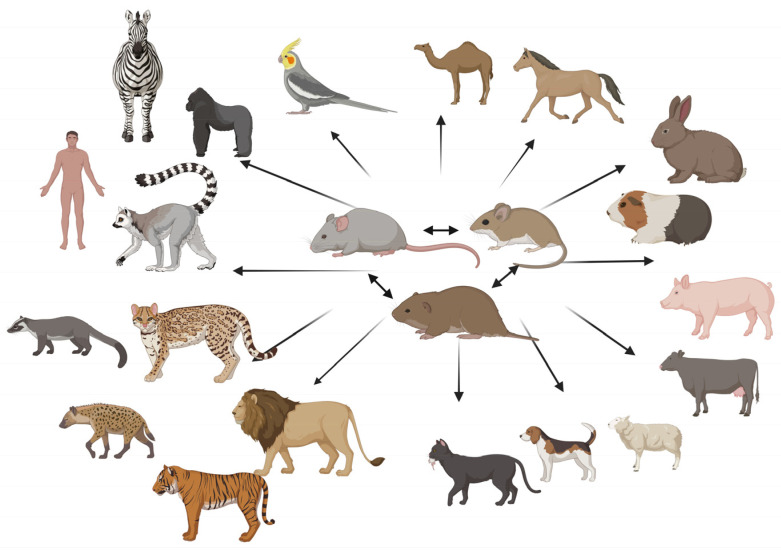
Terrestrial hosts of *Encephalitozoon cuniculi*: Known host range and proposed wild rodent reservoirs for transmission to domestic animals, wildlife, and humans (ref. [9]). Figure created in https://BioRender.com.

*E. cuniculi* is the prevailing species of microsporidia in rabbits [9]. Seropositivity can reach up to 85% by country [19,20,21], and a recent publication suggests a central role of rabbits in the zoonotic transmission of *E. cuniculi* [22]. Nonetheless, domestic and sympatric wild animals, e.g., murids, wild boar and pigs, or horses, have also tested positive for zoonotic genotypes Ec I, II, III, and Ec IV (Figure 1) [18,23,24,25,26]. In addition, contact with birds [27] and consumption of fermented pork contaminated with spores [28] have been reported as sources of zoonotic infection. Moreover, direct contact between immunosuppressed individuals and *E. cuniculi*-infected pets must be considered as a risk factor when specifically vulnerable patients are exposed [29]. These are just a few examples of the broad and complex epidemiology of *E. cuniculi.* They demonstrate that, although rabbits must of course be considered as a source of infection for other species (including humans) [30], they are likely not the only, and probably not even the primary, source of infection, since not only the host range but also contact rates have to be considered in this context.

**Figure 2:**
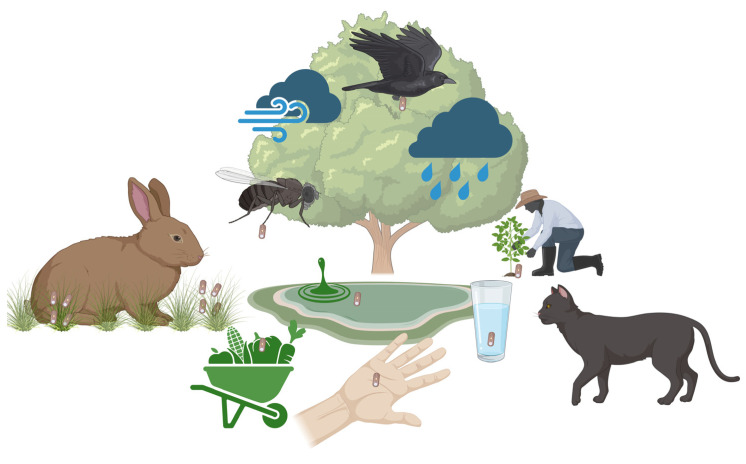
*Encephalitozoon cuniculi * spores excreted by infected hosts via urine, feces, or sputum can be disseminated by soil movement, rain, surface water, coprophilic flies, and other mechanical vehicles as well as predatory animals, supporting a continuous level of environmental contamination with infectious stages. Figure created in https://BioRender.com.

Ingestion of spores can be considered the most likely source of infection for rabbits and humans, since prevalence data on wildlife and domestic animals suggest significant environmental exposure (Figure 2).

Spores are excreted in sputum, feces, or urine and can be disseminated by air or water and consequently contaminate foodstuff or drinking water. At lower temperatures (including frost), spores can survive in water for 12 months or longer, while heat (>60 °C) can destroy them within minutes [31,32]. Presumably, rabbits living in close contact with previously infected animals excreting spores encounter an increased risk of infection with *E. cuniculi*, although data on this are scarce. After ingestion, the parasites can multiply and disseminate in the host organism and transiently infect lungs, liver, and other organs before they settle in brain and kidney epithelia. Infections often either remain subclinical even for a lifetime or cause clinical signs according to the site of infection [33,34]. Notably, infection of dams during pregnancy is considered to result in transplacental transmission to the fetus with the consequence of infection of the ocular lens during the phase of eye development [35]. More recent studies showed that ocular infection can also occur in adult rabbits after oral infection [36].

**Figure 3:**
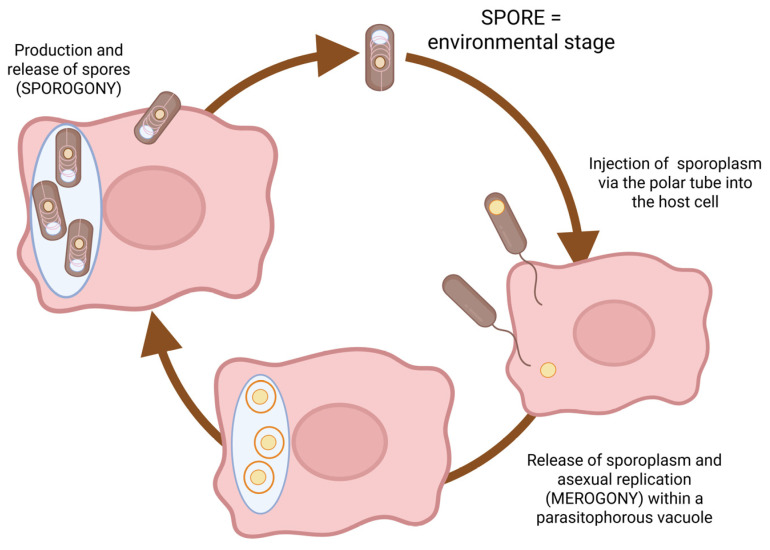
Development of *Encephalitozoon cuniculi* in host cells. Upon ingestion of spores, sporoplasm is introduced into host cells, and asexual replication (merogony) is initiated, resulting in the formation of new spores (sporogony) released into the extra-/inter-cellular space. Figure created in https://BioRender.com.

After ingestion, *E. cuniculi* invades the intestinal epithelium by extruding the polar filament. The sporoplasm is transferred through this filament directly into the host cells, where the *E. cuniculi* multiplies by merogony (asexual multiplication) and sporogony (spore formation). Infectious spores or merozoites (proliferative forms) are then disseminated throughout the body via infected macrophages or by release into the blood stream (Figure 3) [5].

**Figure 4:**
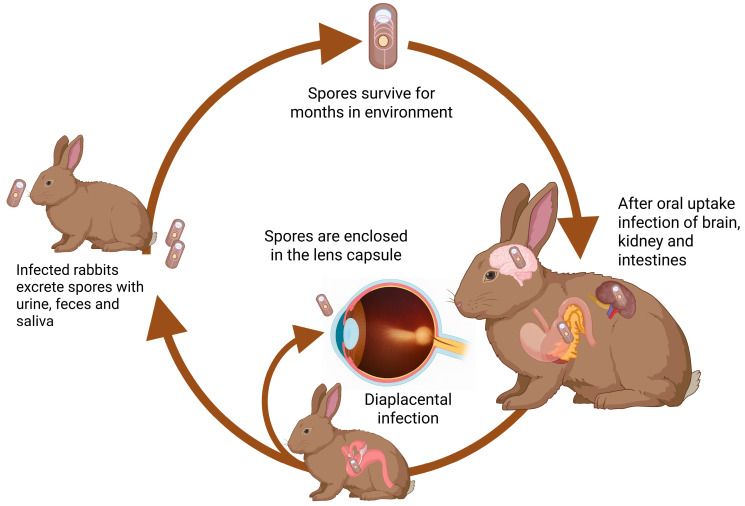
Dissemination of *Encephalitozoon cuniculi* in the infected rabbit and excretion of spores. Horizontal transmission leads to disseminated infection, preferentially in kidney, intestines (followed by excretion of spores with urines, feces, and sputum), and brain, while vertical prenatal infection can lead to enclosure of spores and multiplication in the ocular lens during fetal development. Figure created in https://BioRender.com.

Organs with high blood flow, such as the kidneys, lungs, and liver, are the first target for *E. cuniculi* in rabbits, while the kidneys and the brain are the final predilection sites (Figure 4) [37]. The spread of microsporidia is rapid, and spores can be detected as early as 2 weeks after infection in all affected tissues, including the eye structures and the lens [36,38]. From 35 days p.i., the spores are excreted in the urine of infected rabbits for up to 3 months and then intermittently thereafter, and thus pose a reduced risk to contact animals [37].

With the use of various methods, worldwide seroprevalence in pet rabbits has been reported to range from 41 to 85% [21]. Infection has also been documented in farmed rabbits, and *E. cuniculi* is considered a pathogen in laboratory animal colonies, which has led to concerns that it can affect research findings [21,39]. The high seroprevalence in pet rabbits is similar to the widely held impression of veterinarians, who estimate 30–40% of patients are suspected to be infected [40]. In total, these findings are supportive of the hypothesis that while most infections are subclinical, there is a high prevalence of infections with this organism in rabbits.

## 3. Immunology and Immunodiagnostics

### 3.1. Immune Responses to Experimental Infection

Intravenous, intratracheal, intraperitoneal, intrarectal, intracerebral, and intraocular routes of infection of *E. cuniculi* have been successful in rabbits [37,41,42]. For a detailed characterization of humoral and cell-mediated immunity in rabbits, healthy, immunocompetent adult animals were experimentally infected orally to simulate the natural infection pathway. The immune responses to microsporidia are both cellular and humoral. Despite the subclinical course of the infection, a strong and rapid humoral response was observed. Specific IgM antibodies were detected in the serum of infected rabbits from one-week post-infection and specific IgG antibodies one week later [38,43]. Although the humoral response alone does not appear to be protective, the antibodies contribute to host resistance and have been shown to opsonize microsporidia and thus facilitate killing by macrophages [44,45].

**Figure 5:**
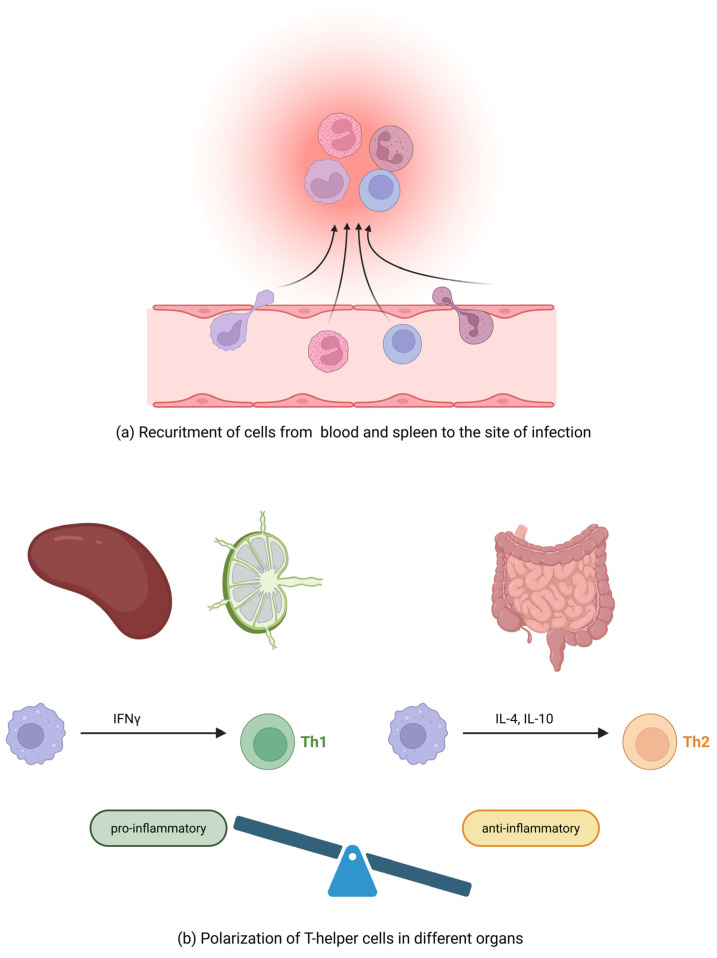
*Encephalitozoon cuniculi* induces tissue-dependent immune responses. (**a**) Upon infection, immune cells are recruited from the spleen and blood to the sites of infection. (**b**) While in the spleen and the kidney (the organ considered to be primarily infected), an interferon-γ-driven proinflammatory (Th1) response prevails, and in the intestines, an anti-inflammatory, interleukin-4 and interleukin-10 (Th2)-dominated response is found [38]. The balance between these two likely determines the outcome of infection. Figure created in https://BioRender.com.

The cell-mediated immune response plays an important role in the prevention of lethal encephalitozoonosis in mice [46]. A proliferation assay was performed on spleen cells to detect the lymphocyte subpopulation responsible for antigen-specific proliferation in orally infected rabbits [38]. Both CD4+ and CD8+ T cells proliferated significantly 2, 4, 6, and 8 weeks post infection (p.i.). The proliferation of CD4+ T cells dominated 2 weeks p.i. In the 4th week after infection, the proliferation of CD4+ and CD8+ cells was comparable and, in the 6th and 8th weeks p.i., the proliferation of CD8+ cells exceeded that of CD4+ lymphocytes. Molecular techniques were used in this study to detect cytokines, another mediator involved in cell-mediated immunity (Figure 5).

A significant increase in interferon-gamma (IFN-γ) mRNA and a polarization of the immune response towards T helper (Th) cell type 1 (Th1) were detected from 2 to 8 weeks p.i. in the spleen, mesenteric lymph nodes, and Peyer’s patches of rabbits orally infected with *E. cuniculi*. In contrast, the predominance of a Th2 cytokine response because of a significant increase in the expression of the Th2 cytokines interleukin (IL)-4 and IL-10 without an increase in IFN-γ mRNA was detected in the small intestine. This may indicate a balanced control of IFN-γ that prevents tissue damage (Figure 5). Although mRNA for IL-17 was found in detectable amounts in all experimental animals, the Th17 lineage appears to play only a minor role during *E. cuniculi* infection in rabbits [38]. These results correspond with the described increase in serum IFN-γ levels in naturally infected rabbits [47]. Similarly, infection with *E. cuniculi* induces a strong cellular immune response in immunocompetent mice, characterized by the production of IFN-γ. Mice unable to produce this cytokine are highly susceptible to infection [48]. There is evidence that IFN-γ, as a proinflammatory cytokine, is a potent activator of macrophages, resulting in the effective killing of phagocytosed microsporidia spores through the production of toxic oxygen metabolites [49].

The comparison of serum proteins of seronegative (presumed uninfected), infected but healthy, and clinically affected rabbits revealed a number of proteins upregulated in the rabbits with clinical signs including antithrombin-III and other markers of immunological processes and coagulation activation, together with proteins signifying the activation of cellular processes, genetic activation and signaling, and cellular metabolism and transport [50]. Another study also found increases in many cellular proteins, including haptoglobin and ceruloplasmin, in rabbits with presumed infection, and in those with neurological signs, acute-phase reactants including hemopexin, alpha-2 macroglobulin, apolipoprotein A-1, and complement proteins were found to be elevated [51].

### 3.2. Serodiagnostic Testing

In addition to the physical examination, serological testing is a primary option for the diagnosis of *E. cuniculi* infection in rabbits [40]. Many methods have been described for the detection and quantitation of specific anti-*E. cuniculi* IgG and IgM antibodies and include the indirect immunofluorescence test (IFT), enzyme-linked immunosorbent assays (ELISAs), carbon immunoassay, and Western blot, although IFT and ELISA are most commonly implemented as diagnostic testing [52,53,54,55]. In all systems, the antigen is prepared from spores harvested from a culture supernatant of *E. cuniculi*-infected cell cultures. While interlaboratory studies have indicated a correlation between assays with respect to the identification of positive sera, it is important to note that quantitative data (i.e., titers) and positive cutoff levels are likely not the same, as the assays are neither equivalent nor standardized across diagnostic laboratories [53,54,55]. For rabbits, there is a single report utilizing Western blot and subsequent mass spectrometry identifying eight antigens, inclusive of polar tube and spore wall proteins, which are targets of the humoral immune response [51,55].

Although serological testing is considered a primary ante mortem tool for many infectious diseases of animals, there are conflicting reports regarding the application of such tests for the diagnosis of disease-related *E. cuniculi* infection given possible acute or chronic presentations. This may stem from the methods that are utilized as well as the case definition of study samples, as definitive post mortem diagnosis is rare. Using IFT and samples from rabbits with suspected *E. cuniculi* infection, as well as from rabbits with other diseases, no difference was found in seropositive status or IgG titers [56]. In contrast, when using a similar group definition and a whole-antigen ELISA, IgG and IgM titers were found to be significantly higher in rabbits with presumed infection versus healthy rabbits and rabbits with other diseases [57,58]. This is inclusive of rabbits that were IgG+IgM+ as well as IgG+IgM−, so the absence of IgM does not rule out active infection.

In an experimental model of infection, IgM was detected within 1 week after inoculation and persisted for at least 4 months, whereas IgG was detectable from 2 weeks p.i. onward [43]. Other models also supported a timeframe of first IgG detection from 10 to 35 days [37,59,60,61]. Over time, IgM titers can fall below the minimum detectable threshold and IgG titers can decrease but rabbits will not become seronegative on whole-antigen ELISA testing (C.C., personal communication). Experimental models of infection have demonstrated decreased titers within 35 days [37]. However, positive IgM titers can be observed in clinically normal pet rabbits with presumed subclinical infection and have also been found to persist in some rabbits previously treated for *E. cuniculi* infection [43,57]. Traditionally, the presence of IgM could be conservatively considered a marker of active infection; however, persistent IgM titers can also be observed with other parasitic infections in the absence of clinical disease [62]. Hypotheses for this persistence include chronic infection, reinfections, or possible derangements of the immune system [62]. With a goal of improving positive predictive value, examination of levels of C-reactive protein, a major acute-phase protein in the rabbit, may aid in demonstrating the presence of a systemic inflammatory process [63,64]. It should be noted, however, that the detection of C-reactive protein is not specific for *E. cuniculi* infection as the acute-phase response can originate from many different etiologies. Acute-phase proteins, when elevated at initial testing, may also prove useful as prognostic indicators when used as a repeated measure [63].

Results of serological testing are related to animal immune competency, circumstances of exposure, and the period prior to seroconversion. Relative to this point is that a few rabbits with lymphoma and very young rabbits have been described to be seronegative in the presence of infection [37,56]. In contrast, in a laboratory animal colony with endemic infection, passive transmission of antibodies was demonstrated from infected rabbits to their offspring [65]. Maternal antibodies were observed through 4 weeks of age, and young rabbits were described to be able to produce antibodies by 8 weeks of age.

Regardless of the methodology, studies agree on the interpretation of a negative serological result. Given the course of infection, seroconversion will occur prior to the time that significant clinical signs are present, so in practice, a false negative result (i.e., disease prior to seroconversion) is unlikely with the exception of the aforementioned factors. Consequently, in clinically abnormal immune-competent rabbits, a negative serological result is reflective of the absence of *E. cuniculi* infection. Also, when serology is used as a screening test (e.g., when animals are tested prior to introduction to a new group), it should be understood that (clinically normal) rabbits may be negative if exposure has been very recent. For rabbits with clinical signs that have been treated, repeated serological testing may provide some information of value. Many rabbits will show decreased IgG reactivity and, with time, negative IgM reactivity, but it should be noted that even successful treatment will not result in a negative IgG result. Variability in post-treatment serological reactivity has been observed, which underscores the need for interpretation of such results with clinical signs and patient history.

## 4. Neurological Presentation

### 4.1. Clinical Overview and Epidemiology

**Figure 6:**
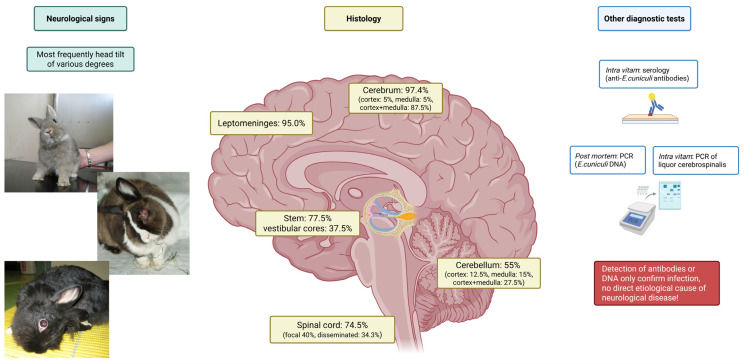
*Encephalitozoon cuniculi* causes disseminated infections in various areas of the brain: pathohistological findings in animals with latent infection versus clinical manifestation [33]. Spores and granulomatous lesions are most frequently found in the cerebrum and leptomeninges, and they do not seem to correlate with the typical clinical sign of head tilt of various degrees. Further diagnostic tests can be performed ante mortem to determine infection and post mortem to establish the presence of spores in brain tissue. Figure created in https://BioRender.com.

Infection with *E. cuniculi* has been recognized for some time in laboratory rabbits and has been identified as a significant cause of neurological disease in pet rabbits worldwide over the last 20 years [52,66,67,68,69,70]. Following infection with this parasite, the host cell eventually ruptures, releasing spores into the extracellular space and resulting in chronic diffuse cellular infiltration and granuloma formation in target organs such as the brain [71]. Histologically, lesions within the central nervous system (CNS) occur at least one month p.i. [72]. Csokai et al. reported that nonsuppurative or granulomatous encephalitis associated with *E. cuniculi* infection was most found affecting the cerebrum, brain stem, and less so the cerebellum, with the vestibular cores being least affected (Figure 6) [33]. Pathological changes do not always correlate with clinical signs, and severe brain pathology can be found in clinically normal animals [33]. Brain lesions observed include perivascular cuffing, meningitis, and large granulomata formation, resulting in a nonsuppurative granulomatous meningoencephalitis [72]. The inflammation will commonly form glial nodules composed of microglia and astrogliosis, which form around *E. cuniculi* organisms [73]. These organisms reside in parasitophorous vacuoles located within macrophages/microglia and endothelial cells or are ruptured and found within the neuropil. This organism stains poorly with hematoxylin and eosin stain, and special stains are often required to identify the *E. cuniculi* spores within the lesions. The best stains have been shown to be a Gram’s stain (*E. cuniculi* is Gram-positive) or modified trichrome stain when using light microscopy, and calcofluor white stain when using ultraviolet light microscopy [74]. The MTS stain helps visualize specific spore structures (posterior vacuole, polar tube, and polaroplast) to more definitively identify that the spores are *E. cuniculi* [4,74,75].

In addition to *E. cuniculi*, potential recognized causes of primary CNS disease in any mammalian species include infectious agents, inflammatory diseases, neoplasms, traumas, congenital and degenerative diseases, metabolic diseases, and toxins. Confirmed infectious causes in rabbits include rabies, human herpes-1 virus, and *Baylisascaris* sp. larva migrans [34,76,77,78,79]. CNS neoplasia is rare in rabbits. A single case of glioblastoma was reported in a rabbit on necropsy [80]. The most recognized infectious causes of CNS disease in rabbits include bacterial otitis media/interna and *E. cuniculi* infection, both of which can present with similar clinical signs and can occur simultaneously [81]. It should be noted that experimental models of these mechanisms in rabbits are lacking.

The exact mechanism whereby *E. cuniculi* infection leads to the development of these signs is not clear, and encephalitis is often described, rather than labyrinthitis or vestibular neuritis [33,82]. In rabbits with clinical signs, vestibular disease is most commonly observed as a potential secondary effect of granulomatous inflammation in the brain [33,34,43,83]. In one retrospective pathological study of rabbits with neurological disease, 58.5% of rabbits were diagnosed with *E. cuniculi* [83]. In another study, 77.1% of rabbits showing neurological signs were seropositive for *E. cuniculi* [34]. Seropositive rabbits with neurological signs had a mean age of 2.8 years [34].

Clinical signs of vestibular disease include circling, falling to one side, rolling, head tilt, ataxia, and nystagmus (Figure 6) [33,34,68]. Vestibular signs may range in severity from mild to extreme, may respond to minimal treatment and resolve within a week, or may last for many weeks or months [34]. In this retrospective study involving 184 rabbits, 54.2% of the patients exhibiting neurological signs recovered within a few days and typically showed no reduction in appetite [34].

Other neurological signs anecdotally reported with this parasite include hindlimb paresis, seizures, urinary incontinence, and bladder distension [67,68]. However, these signs sporadically associated with *E. cuniculi* infection need to be critically evaluated, as others (traumatic lesions such as luxation or fracture, neoplasia, or spondylosis) may be more likely causes [33,34].

Concurrent infections with *E. cuniculi* encephalomyelitis and bacterial otitis media/interna have been reported in cases presenting with head tilt [43,68,81,83]. Other reported concurrent infections in pet, farmed, and wild rabbits include *Toxoplasma gondii*, *Neospora caninum*, and herpes simplex virus infections [83,84,85,86]. Comorbidities such as malignant lymphoma, bacterial encephalitis, hepatic cirrhosis, and hydrocephalus may also occur [33,83].

### 4.2. Physical Examination

Vestibular disease is characterized by clinical signs that can include head tilt, nystagmus, ataxia, and circling, and severely affected rabbits can be non-ambulatory and in lateral recumbency or even rotating along the body length axis. This way of rotating is unusual, and it might be misinterpreted as seizures by some animal owners. Nevertheless, seizures in rabbits with *E. cuniculi* have been anecdotally described (E.K. and J.G., personal communication).

Typically, vestibular signs have a sudden onset and often follow a stressful event [34]. The neurological examination in rabbits is carried out in the same way as in dogs and cats. However, some portions of the exam are less useful in rabbits, for example, the menace response. A modified neurological examination has been suggested for this species and includes minimizing restraint and handling stress, performing portions of the exam in the rabbit’s enclosure where possible, covering the eyes to reduce visual stimulation, and using gentle and non-threatening approaches [79,87]. Observation of spontaneous behaviors and gait prior to handling is often more informative than provocative testing, as exaggerated restraint or startling maneuvers can lead to stress-induced collapse or injury. Careful evaluation may enable a differentiation between central (vertical or positional nystagmus, further cranial nerve deficits, depressed mental status, cerebellar signs, or postural reaction deficits) and peripheral vestibular disease. Although, *E. cuniculi* causes multifocal meningoencephalitis, affected rabbits almost always show only signs of peripheral vestibular disease instead of central vestibular disorders that could be expected in rabbits with encephalitis caused by *E. cuniculi* [34]. It is not understood why rabbits mainly show uniform clinical signs in the form of vestibular disorders, despite histological alterations that are characterized by multifocal pathohistological lesions within the brain and spinal cord. In principle, there are several differentials for vestibular dysfunction in rabbits, including other infections, head trauma, neoplasia, as well as drug-induced disorders and intoxications [83]. However, otitis media/interna represents a main cause of vestibular disorders that must be differentiated from encephalitozoonosis [81,88]. Specific clinical signs, i.e., facial nerve deficits in terms of facial nerve spasticity, a clear indication of otitis media/interna, are helpful in the differentiation from *E. cuniculi* infection [79,89]. In contrast to rabbits with head trauma, food intake is commonly not reduced, even in severe cases of vestibular dysfunction due to encephalitozoonosis [34]. However, it must be considered that if animals are in lateral recumbency and unable to sit up, they are not able to reach their food. This can be misinterpreted as inappetence caused by nausea. Other clinical signs associated with severe or longer-lasting vestibular disorders include injuries to the eye, most commonly on the side of the head tilt, but also affect the uppermost eye and decubital sites. The physical examination should include fluorescein staining of the ocular cornea. Rabbits with an increased degree of head tilt show rolling more often, resulting in an inability to consistently sit upright and a position of lateral recumbency. A prolonged period of immobilization due to an acute vestibular disorder may limit the recovery from vestibular dysfunction [90].

### 4.3. Diagnostics

Routine diagnostic bloodwork is sometimes helpful to rule out differential diagnoses for suspected CNS disease in rabbits, including sepsis, hypoglycemia, hypocalcemia (which is rare in rabbits), and organ failure. Pathogen testing could be considered for specific suspected infectious etiologies. A routine complete blood count (CBC) and biochemistry analysis are useful for an overall assessment of general health and should be part of any workup for CNS disease in rabbits. Diagnostic imaging for suspected neurological expressions of *E. cuniculi* is mostly helpful for ruling out other disease processes but is not useful for a definitive diagnosis. Modalities described include radiology, computed tomography (CT) imaging, and magnetic resonance imaging (MRI), and even ultrasound [91,92]. Each modality can be enhanced with the use of contrast. Imaging is of critical importance for recognition of otitis media, and for some modalities, interna [93]. Radiographs are less sensitive for otitis media but will often detect thickening of the wall of the osseous bullae, or bony expansion and destruction in advanced cases. However, CT scanning is far superior and will demonstrate both bony changes and fluid filling of the bulla. For the detection of abnormalities related to the otitis interna, MRI is considered superior [91]. Ultrasound was also investigated for the detection of fluid filling of the osseous bulla and found useful [92]. Imaging can also help to rule out other less common etiologies including trauma and neoplasia; MRI has been found useful in humans to rule out parasitic neural larval migrans and may be useful in other species as well [94]. Contrast is used to enhance imaging for evaluation of the CNS [95]. The use of contrast is described in more detail in the renal imaging section of this document. Contrast is ideally administered via a cephalic vein intravenous (IV) catheter; however, use of the lateral saphenous and marginal auricular veins has also produced satisfactory results [95]. Dosages are extrapolated from other pet species. One author (A.L.) uses iohexol injection at 3–4 mL/kg IV over 2 min. In conclusion, while it is not possible to make a definitive diagnosis of *E. cuniculi* using imaging modalities, they are very useful in determining differential diagnoses.

### 4.4. Treatment

#### 4.4.1. Conventional Treatment

Clinical disease is challenging to diagnose and treat effectively. To reduce inflammation, a reduction in spore proliferation and migration appears an important component of treatment. Additional goals include the management of concurrent diseases and severe neurological signs. Current treatment protocols for neurological presentations focus on a multimodal approach including anti-microsporidial agents, antibiotics for concurrent infections, anti-inflammatories, benzodiazepines and vestibular support medications, and supportive care (Table 1). Based on antibiotic stewardship, antibiotics are only indicated where there is evidence of a bacterial agent, and antibiotic choice should be determined by culture and sensitivity results. Antibiotics are not indicated for the treatment of *E. cuniculi* but may be indicated where there is evidence of concurrent bacterial infection.

- Anti-microsporidial therapy

Benzimidazole anthelmintics have demonstrated efficacy against *E. cuniculi* in vitro and have been effective to prevent and treat naturally acquired and experimental *E. cuniculi* infections in rabbits as well as help prevent infection in immunosuppressed rabbits [96,97]. Long-term treatment for 30–60 days is often recommended, as it is believed this drug primarily inhibits parasite replication rather than eliminates the organism entirely, and spores remain in the environment long-term [21,79,82]. The effectiveness in rabbits already exhibiting clinical signs remains uncertain. While no formal study has addressed this issue, it has been anecdotally voiced that some rabbits with neurological signs improve over time with supportive care and medications exclusive of benzimidazoles (F.K. and S.K., personal communication).

Among the available treatments, fenbendazole is the most used and has shown the greatest effectiveness [21,79,82]. Like all benzimidazoles, fenbendazole inhibits β-tubulin polymerization, which disrupts microtubule formation and impairs parasite replication. Additionally, it has been shown to reduce inflammatory cytokines in other species: IFN-γ, TNF-α, and IL-1β [98]. Current dosage recommendations include a 28-day treatment with fenbendazole (20 mg/kg PO q24 h) or oxibendazole (15 mg/kg PO q24 h) [97,99]. Albendazole is an alternative treatment but is less often used due to a higher risk of hepatotoxicity and embryotoxic effects [100].

**Table 1:** Common medications for neurologic disease in rabbits [99,101,102].

Category	Drug	Dose	Indication
Anti-microsporidial	Fenbendazole	20 mg/kg PO q24 h × 28 d	Microsporidial infection
	Oxibendazole	15 mg/kg PO q24 h × 28 d	Microsporidial infection
Antibiotic	Enrofloxacin	10 mg/kg PO q12 h	Concurrent bacterial infections
	Trimethoprim-sulfonamide	30 mg/kg PO q12 h	Concurrent bacterial infections
	Chloramphenicol	50 mg/kg PO q12 h	Concurrent bacterial infections
NSAID	Meloxicam	0.5 mg/kg PO q12 h, up to 1 mg/kg PO q24 h	Neuroinflammation
Anticonvulsant	Midazolam	0.25–1 mg/kg SC/IM/IV q6–12 h PRN	Acute seizure control
	Diazepam	1 mg/kg IV, then 0.5–2 mg/kg PO q8–12 h *	Chronic seizure management
	Gabapentin	5–10 mg/kg PO q12 h, up to 25 mg/kg PO, q24 h	Seizure and pain management

* Personal communication (S.K.). Key: mg = milligram, kg = kilogram, q = every, PO = per os, SC = subcutaneous, IM = intramuscular, IV = intravenous, h = hour, d = day.

The use of benzimidazoles is not without risk as bone marrow suppression and fatal pancytopenia in rabbits are described sporadically [103]. Ideally, the implementation of a CBC prior to initiating benzimidazole therapy, as well as 7–10 days after starting treatment, to detect any early signs of bone marrow suppression is recommended. Stopping benzimidazole treatment if pancytopenia is noted may result in partial or full recovery in some rabbits (J.G., personal communication). If bone marrow suppression does occur, emergency treatment including blood transfusion, erythropoiesis-stimulating agents such as epoetin alfa (50–150 U/kg SC q2–3 d), or filgrastim to stimulate neutrophil production (5 mcg/kg SC q12 h) may be beneficial in specific cases [99]. It should be noted that these treatment options are extrapolated from their use in dogs and cats, and the efficacy and side effects have yet to be fully evaluated in rabbits. Unless it is detected early, the prognosis for the recovery of bone marrow suppression is poor.

- Antibiotic therapy

In addition to *E. cuniculi*, otitis media/interna is a common cause for neurological signs in rabbits. These conditions may occur concurrently [81]. When otitis is suspected, antibiotics are often used to manage neurological signs in rabbits, but their use should ideally be based on a definitive diagnosis obtained using CT imaging of the bullae (A.L. and F.K., personal communication). Selecting an appropriate antibiotic for rabbits requires careful consideration of their unique hindgut physiology to avoid antibiotic-induced dysbiosis. Medications must be chosen with a focus on safety and efficacy in lagomorphs. Culture and sensitivity testing may be helpful. Suitable systemic antibiotics include enrofloxacin (10 mg/kg PO q12 h) and trimethoprim-sulfonamide (30 mg/kg PO q12 h), both of which have CNS penetration and are well-tolerated and effective against common bacterial infections in rabbits [99]. While chloramphenicol (50 mg/kg PO q12 h) is a broad-spectrum drug highly effective for CNS infections, it can cause bone marrow suppression in humans and is used less frequently as a result [99]. Antibiotic choice should be based on the principles of good stewardship with an aim to minimize the development of resistance.

- Anti-inflammatory therapy

It is reasonable to assume that chronic inflammation contributes to neurological signs in *E. cuniculi*-infected rabbits. As such, non-steroidal anti-inflammatories (NSAIDs) can be a useful supplement to treatment. Meloxicam (0.5 mg/kg PO q12 h; up to 1 mg/kg q24 h) is the preferred NSAID due to its safety profile and ability to reduce neuroinflammation, although caution should be used in patients with impaired renal function [99,104]. A recent study evaluating otitis media/interna and encephalitozoonosis in rabbits in the UK found that no one treatment was significantly associated with an improvement in otitis media/interna cases. However, NSAIDs were significantly associated with higher chances of improvement in *E. cuniculi* cases [81]. Steroid therapy should be avoided as it is associated with a risk of intestinal and liver problems, along with immunosuppression in rabbits. Additionally, steroid therapy showed no therapeutic effect in chronically infected rabbits [105].

- Anti-convulsant and vestibular support therapy

Benzodiazepines are a frequent treatment for dizziness and vertigo in humans [106]. Midazolam and diazepam have been used to manage signs associated with head tilt, and rolling in rabbits. Midazolam (0.25–1 mg/kg IM, SC, IV q6–12 h PRN) can be titrated to effect, with doses lower than 0.25 mg/kg used by one author (S.K., personal communication), to calm rolling rabbits without sedative side effects. It also helps reduce anxiety and aid in relaxation in rabbits with vestibular signs [99,107]. This drug also acts as an appetite stimulant in rabbits. Diazepam can be used as an alternative at 1 mg/kg IV for severe cases with rolling. Oral diazepam (S.K. personal communication), 0.5–2 mg/kg PO q8–12 h, can be used as signs improve, but rolling is still present. Gabapentin (5–10 mg/kg PO q12 h) may reduce neuropathic pain and seizure activity, even if these signs may seem to play a minor role in the disease. A single high dose of gabapentin at 25 mg/kg PO has been shown to reduce stress in rabbits and could be used to reduce rolling in selected cases [108].

Antiemetic drugs such as metoclopramide, prochlorperazine, and meclizine (12.5–25 mg/kg PO q8 h) can be used in cases of severe torticollis, but their effectiveness remains ambiguous, with some clinicians being firm advocates of these medications and others no longer recommending them. Doses have been published in pet rabbits but without pharmacokinetic studies to support the two latter drugs [99]. In addition, maropitant could be considered for its anti-nausea effects (as demonstrated in other species) at 1 mg/kg SC q24 h [109].

#### 4.4.2. Supportive Care and Alternative Treatments

During the active phase of infection with *E. cuniculi,* rabbits will present with vestibular signs such as head tilt, nystagmus, and rolling. Often, rabbits are anxious due to disorientation, and consequently food intake may be reduced in some cases. In addition to conventional medical treatments, there are many supportive measures that can be instigated to keep rabbits comfortable and aid recovery. Despite the lack of controlled clinical trials, supportive care and complementary therapy form an important part of the treatment of rabbits with neurological disorders.

- Hydration and nutritional support

It is challenging for rabbits with vestibular signs to prehend food. They may also experience vertigo and nausea, which could suppress their appetite in some cases. Within a few days, rabbits usually learn to adapt to the vestibular signs, but until then, they require supplemental hydration and nutritional support [79]. Subcutaneous or intravenous crystalloids should be administered (maintenance rates 2–4 mL/kg/h IV or 50–100 mL/kg/day SC in several sites, divided q6–8 h) until the rabbit is eating, urinating, and defecating well [110]. Hydration is especially important if there is any underlying renal disease and allows the rabbit to safely be treated with NSAIDs (see renal section for calculation of fluid deficits). Rabbits with significant rolling should not have a water bowl or bottle left in their cage, as they may injure themselves. Alternatively, water can be offered to them several times a day. In some cases, assisted feeding is required and will provide nutritional support, as well as some hydration. It is important that care be taken when syringe feeding cases with head tilt, given the position of the head, since the rabbit is at increased risk of aspiration and nasal reflux. It is recommended to introduce only a few milliliters of food at a time via the diastema into the oral cavity and observe closely for swallowing reflexes. Rabbits should be syringe fed with a commercially available formula such as Critical Care for Herbivores (Oxbow Pet Products, Murdock, NE, USA), Emeraid Sustain (Lafeber Company, Cornell, IL, USA), Recovery (Sherwood Pet Health, Ephraim, UT, USA), or Science Selective Recovery Diet (Supreme Pet Foods, Ipswich, Suffolk, UK). A general guideline is 10–20 mL/kg of the mixed product orally every 4–8 h until eating well [110]. Rabbits should be fed frequently with large amounts of leafy greens during their illness, and these should be placed immediately in front of their mouth at all times or fed by hand. Hay and pellets can be offered, and as the vestibular signs subside, rabbits are more likely to eat these as well. If a rabbit has severe rolling and spends a lot of time on their side or in dorsal recumbency, the greens can still be placed near their mouth (S.K. personal communication).

- Housing and restraint

Housing is adjusted based on the severity of the vestibular signs, which can change over time. All rabbits with vestibular disease benefit from padded cage sides, secure non-slip footing, and foot and water arranged to be readily accessible, and not a danger should the rabbit roll into it. In most cases, all other cage furnishings and toys should be removed. Rabbits that can still ambulate and use a litter box may benefit from a shallow box, or one with one side cut down to make entrance and exit easier. Rabbits that are constantly rolling will benefit from more significant padding and support [88,111]. A small cage or large top opening carrier can be used. The carrier should be padded with rolled-up towels on each side. The towels should extend high up the sides to provide a deep narrow “canyon” for the rabbit to reside. This gives them support to lean against and a light pressure on both sides to decrease rolling. A towel or other absorbent bedding, placed on the bottom of the carrier, will provide soft footing, reduce pressure sores, and should be changed frequently since affected rabbits are unable to use a litter tray and tend to urinate and defecate where they are sitting. This can lead to the development of urine scald and resulting perineal dermatitis, as well as increase the risk of myiasis. Regular inspection and cleaning of the perineum are also therefore required. Rabbits may still roll using this setup, but the rolling is lessened and more controlled. It is acceptable if the rabbit spends most of the time lying in dorsal recumbency as they may feel more secure, with reduced spinning sensation, in that position until the vestibular signs diminish. Rolling usually improves over time, and housing can be adjusted to adapt to the rabbit’s condition. Rolling rabbits are likely to spill water and food. While some can be left with food within easy reach, others may need to be offered food and water directly, with amounts based on the rabbit’s ongoing needs. For further information, refer to the Housing Video (see Supplementary Materials Video S1).

When a vestibular rabbit is lifted and moved, they will often struggle, and clinical signs worsen. It may be advantageous to administer medications and treatments with the rabbit remaining in the carrier or cage (refer to the Medication Video, Supplementary Materials Video S2). The exception to this is when performing physiotherapy, which can start immediately and is an important treatment component in the early stage of vestibular disease, especially in severe cases (F.K., personal communication, see alternative treatments). It can take two weeks or longer for vestibular signs to improve. The rabbit can be hospitalized for this time, or owners can also be taught to care for these rabbits, but rabbits with severe rolling usually need round-the-clock care (S.K., F.K., personal communication). Another option is to have the rabbit cared for by a technician or an experienced rabbit rescuer until the signs subside. If the owner opts for caring for their pet, they should be taught how to lift them to prevent rolling (refer to the Handling Video, Supplementary Materials Video S3). It is recommended to lift the body under the chest with one hand and use the other hand to hold the head in line with the body. The rabbit’s body should be brought close to the owner’s body for additional support. Then, place all four feet down on a solid padded surface while still holding onto the head and body, until the rabbit is standing securely. A supportive hand should always be kept on the rabbit.

As the rolling subsides, the rabbit can have less padding and a larger area. At that point, more exercise and physical therapy are a major part of the treatment to help the rabbit ambulate better and aid recovery.

Rabbits that are severely rolling and are bonded to another rabbit should be separated. They can have visitation time with the bond mate. If the vestibular signs are mild, then they can remain with their bond mate, although monitoring of food and water intake and fecal and urinary output of the individual affected animal is more challenging.

- Ophthalmologic trauma treatment

Rabbits that are falling over or rolling often develop corneal ulcerations and conjunctivitis; therefore, fluorescein corneal staining should be performed as part of the physical exam. At the onset of signs, it is recommended to apply a lubricating eye gel such as Artificial Tears (Henry Schein, Melville, NY, USA), Systane (Alcon Laboratories, Fort Worth, TX, USA), Optixcare (Aventix, Burlington, Canada), VisuEvo Ophthalmic solution (Visufarma, Amsterdam, The Netherlands), or Hyabak (Thea pharmaceuticals Ltd., Newcastle, UK) to both eyes every 6–12 h. If a corneal ulcer or conjunctivitis develops, then the rabbit should receive an antibiotic ophthalmic medication every 6–8 h and continue to receive the lubricating gel with at least a 15 min time lapse between treatment applications.

In cases of severe conjunctivitis or blepharitis, a temporary tarsorrhaphy on the affected eye may help protect the cornea and facilitate healing. Alternatively, studies in rabbits have demonstrated that therapeutic soft contact lenses can aid antibiotic delivery without compromising efficacy, and collagen shields can accelerate epithelial healing in corneal injuries [112,113]. Collagen shields have also been successfully applied in clinical cases of chronic corneal ulcers in rabbits [114]. Additionally, to improve lens retention, fixation of the nictitating membrane has been shown to significantly enhance contact lens stability [115].

- Alternative treatments

Acupuncture, cold laser therapy, and physical therapy may help decrease the severity of vestibular signs and may speed healing. Stimulating specific points on the body with acupuncture needles or acupressure can improve blood flow to the inner ear, reduce inflammation in the muscles and nerves associated with balance, and regulate the function of the vestibular system. Acupuncture can also help relax tense muscles in the neck and shoulders, and it triggers the release of endorphins and nor-epinephrine, which have pain-relieving and calming effects [116,117,118,119]. Single case reports have been documented in rabbits; however, controlled studies have not been conducted [118].

There is also no data on the use of low-level laser therapy (LLLT) in rabbits with vestibular signs. LLLT is used in many species to improve tissue blood flow and oxygenation, reduce inflammation, and stimulate healing, and is most commonly used to treat musculoskeletal injuries and osteoarthritis [120,121]. When rabbits have a head tilt and are rolling, their neck can be significantly curved or twisted. LLLT may help with the musculoskeletal effects on the neck and may also help decrease inflammation of the vestibular nerve.

Physical therapy may be the most helpful alternative therapy in these cases, with recent recognition of its positive impact on the recovery of rabbits with vestibular disease, allowing an earlier return to normal function [88]. Physical therapy should be started early in the course of the disease to preserve a normal range of motion, challenge nervous system deficits, and strengthen the musculoskeletal system. Rabbits with acute severe vestibular disorders may benefit from hospitalization. Inactivity or immobilization in the early stages may slow or impair recovery. Housing adjustments should be made to help maintain a more normal upright position. In the early stages, simple massage of the neck muscles may be helpful and is well tolerated by the majority of cases.

Physical therapy exercises vary depending on the severity of the vestibular deficits (refer to Physiotherapy Video, Supplementary Materials Video S4). These can include helping the rabbit to maintain an upright sitting position, head support to reduce the degree of head tilt, and encouragement of the rabbit to perform normal, simple limb movements. Passive and active movements of the head will stretch the musculature to prevent contracture, which occurs rapidly with disuse and hinders recovery. The head is gently moved away from the side of contracture to provide a full range of motion. For rabbits unable to stand or ambulate, use of the forelimbs is encouraged by supporting the body and moving the rabbit forward and side to side. Each physiotherapy session may take several minutes and be repeated multiple times daily, adjusted over time depending on the patient’s response (positive or negative).

Once rabbits can sit upright, daily exercise on a non-slip surface is encouraged. Supervision is necessary to help provide immediate support should the rabbit begin to roll. Affected animals usually learn to move in straight lines instead of walking in circles, and the tilt of the head during movement usually becomes less pronounced within a short period of time.

As the rabbit improves, the frequency and length of the exercise sessions can be increased. While most rabbits improve with physiotherapy, residual deficits (mostly minor head tilt) may persist. Many rabbits learn to adapt to these deficits with a good to excellent quality of life.

Humans with vestibular signs may be diagnosed with benign positional vertigo, a condition thought to be caused by otoconia or crystals in the semicircular canals. Some veterinarians include this as a differential diagnosis for vestibular signs in rabbits and have utilized a modified Epley maneuver to attempt to move the otoconia out of the semicircular canals and back into the inner ear. It should be noted that these reports are anecdotal only and reflect variable success rates.

- Client support and prognosis

It is recommended to provide extensive support and education to the rabbit owner. When rabbits first develop neurological signs, it is often acute and distressing for the owner. Owners may think the rabbit is having a stroke or seizing. Owners should be directed to place the rabbit in a well-padded carrier right away to control the rolling and avoid handling the rabbit where possible. They can be reassured that this syndrome is relatively common in rabbits and in general has a good prognosis if treated early. *E. cuniculi*-associated neurological signs can vary from slight to severe and can take a few days to several weeks to improve. The severity of clinical signs is often not correlated with the prognosis, and there is usually no medical reason to euthanize these rabbits initially. However, if the animal is losing weight, shows no signs of improvement, and there are significant welfare concerns, then euthanasia should be considered.

In many cases, the clinical signs will improve or resolve. Some rabbits will have a persistent head tilt but can adapt and live with a good quality of life. Additionally, some rabbits may have repeat neurological episodes, especially after major surgery, illness, or other stressors, as well as when they are geriatric and their immune system is compromised. Conversely, however, some rabbits never have any additional neurological episodes. This needs to be conveyed to the owner to manage their expectations accordingly. In contact, rabbits should be carefully monitored for clinical signs associated with *E. cuniculi* infection; however, since antibody titers can remain elevated for prolonged periods and latent infection is common, serological testing or treatment of clinically healthy in-contact rabbits cannot generally be recommended based on current knowledge.

## 5. Renal Presentation

### 5.1. Clinical Overview and Epidemiology

Both acute and chronic renal diseases are seen frequently in rabbits, especially chronic forms in older patients. Documented etiologies include urolithiasis, toxins, neoplasms, trauma, and infectious diseases, including *E. cuniculi* [122,123]. In many cases, the exact etiology is unknown, and *E. cuniculi* should be considered part of the differential diagnosis.

A survey of 2583 rabbits (pet, laboratory, and production) submitted to diagnostic laboratories showed a 13% incidence of renal disease, mostly in pet rabbits [124]. The most common abnormality was nephritis of unknown origin (68.3% of pet rabbits and 78.6% of meat rabbits). Chronic renal disease including infarcts was seen in 20.2% of pet rabbits. *E. cuniculi* infection was confirmed mostly in pet rabbits and represented 6% of pet rabbits with renal disease [124]. In most cases, lesions of the kidney were not associated with specific disease conditions and occurred with other body system lesions as well.

**Figure 7:**
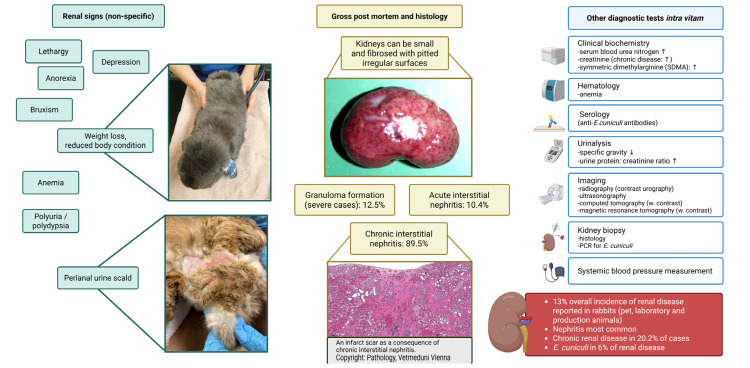
*Encephalitozoon cuniculi* infects the kidneys and causes various renal alterations during the acute and chronic phases of infection that can lead to often non-specific signs of disease. Determination and monitoring of renal function are important to evaluate the full extent of disease. Histological changes as previously described [33] and case incidence as summarized in reference [124]. Figure created in https://BioRender.com.

In addition to the CNS and the eye, the kidney is also a predilection site for *E. cuniculi* (Figure 7) [33,34]. Chronic interstitial nephritis occurs, which in most cases remains clinically latent and only in the advanced stage of infection can lead to renal failure. Such clinically manifested kidney problems are only observed sporadically and are therefore much less common compared to neurological manifestations [124]. Chronic renal failure (CRF) most likely occurs when other cofactors, such as nephrocalcinosis or nephrolithiasis, are present simultaneously, further damaging the kidney. Cases of *E. cuniculi*-induced CRF can present with nonspecific clinical signs like those seen in dogs and cats (e.g., polyuria/polydipsia or chronic weight loss) [52]. Often, renal failure is only detected incidentally during blood or urine analysis (E.K., personal communication). If, in an advanced stage of the infection, the kidney is damaged, the prognosis is generally considered poor [34].

The early histological renal lesions of an *E. cuniculi* infection are composed of multifocal to segmental granulomatous interstitial nephritis associated with renal tubular epithelial cell degeneration, necrosis, and a mononuclear cellular infiltration [74]. These lesions can be present at all levels of the renal tubule and uncommonly involve the glomeruli. If present, the spores can be identified within parasitophorous vacuoles in the tubular epithelium, within macrophages, or free within tubular exudates [122]. For acute lesions, it may be possible to identify the spores using a Gram or modified trichrome stain [72,74]. The spores are Gram-positive, and they are approximately 1.5 × 2.5 µm in size. Chronic lesions consist of a fibrosing interstitial nephritis, which indicates previous or persistent infections (Figure 7). These areas of fibrosis lead to loss and contraction of parenchyma with variable amounts of lymphocytes, histiocytes, and plasma cells. Within these chronic lesions, microbes are seldom identified, even with special stains, although immunohistochemistry may prove helpful [36].

### 5.2. Physical Examination

A full clinical history and examination is important in rabbit cases suspected of renal disease associated with *E. cuniculi* infection, to rule out potential comorbidities, as well as identify any clinical signs associated with renal disease [123]. In particular, ophthalmic and neurological examination as well as gait assessment should be completed, due to the predilection for target organs such as the brain, kidneys, and eyes, despite infrequent observation of combined signs [30].

Clinical signs of renal failure can be non-specific [33] and may include lethargy, depression, anorexia, teeth grinding, weight loss, reduced body condition, anemia, polyuria, polydipsia, and perineal urine scald (Figure 7) [34,52,125]. Assessment of the daily water intake, if polydipsia is suspected, should be performed, with the normal daily water intake in the rabbit reported as approximately 100–120 mL/kg/day and normal daily urine output being 130 mL/kg/day [126,127,128,129]. Older rabbits with a poor body condition and weight loss should be investigated further for possible underlying renal disease [52,88].

Most rabbits do not usually resent gentle abdominal palpation, and repeated flinching, associated with palpation of the kidneys, is likely to be associated with pain [123]. However, chronic renal insufficiency associated with *E. cuniculi* infection can only rarely be detected on clinical examination. Rabbit kidneys are relatively easily palpated on routine clinical examination [130], except in obese animals where they may have excessive peri-renal fat stores [131]. They are retroperitoneal, moderately mobile, smooth, and bean-shaped, with the right kidney being slightly bigger and situated more cranially than the left [132]. The left kidney is more mobile and situated in the mid abdomen, at the level of the second to the fourth lumbar vertebra [133]. The caudal pole of the right kidney is palpable just under the ribs on the right side of the abdomen, at the level of the first lumbar vertebra [123,133]. In rabbits with chronic renal changes associated with *E. cuniculi* infection, kidneys are often small and scarred, with pitted, irregular surfaces (Figure 7) [68,123]; however, this is not always appreciable on palpation during clinical examination. In other studies, gross lesions of kidneys affected by *E. cuniculi* included alterations in size and color, and fibrosis (determined by capsule adherence to renal parenchyma and subcapsular pitting). Histopathologic lesions associated with *E. cuniculi* included chronic interstitial nephritis (89.6%), acute interstitial nephritis (10.4%), and granuloma formation in severe cases (12.5%) [33] (Figure 7). Organisms are not always present in patients with CRF, but may be detected in urine during acute phases of infection [82].

In some cases, kidneys may be enlarged [67]. Evaluation of secondary clinical signs with regular recording of the body weight and body condition score, as well as evaluation for anemia and perineal urine staining, can aid the diagnosis of renal disease [123]. Indirect measurement of systolic blood pressure might be useful to rule out hypertension, which may be associated with chronic renal failure [134,135].

### 5.3. Diagnostics

The diagnostic workup for kidney disease in the rabbit is essentially the same as for other species.

#### 5.3.1. Blood Biochemistry and Renal Function Analysis

Alongside serodiagnostic testing for *E. cuniculi* antibodies, routine diagnostic bloodwork for renal evaluation can be useful in late-stage kidney disease. Standard renal parameters include blood urea nitrogen (BUN) and creatinine, and reference intervals have been determined for domestic rabbits [135]. Neither are considered sensitive for detection of mild or early kidney disease.

Symmetric dimethylarginine (SDMA) has been shown to be a reliable early indicator of chronic renal disease in other animals. Reference intervals for SMDA were established for rabbits; however, correlation with actual disease states remains to be determined [136].

A study evaluated the relationship between *E. cuniculi* serodiagnostics and BUN and creatinine in clinically healthy rabbits. ELISA testing showed that 49% of healthy test rabbits were seropositive, and BUN and creatinine levels were significantly elevated in seropositive animals. Only two rabbits underwent necropsy, and degenerative changes and *E. cuniculi* spores were confirmed in the renal tubule epithelium. While BUN and creatinine were higher in the seropositive animals, they were still within established reference intervals, making clinical utility uncertain [137].

In another study, reference intervals for urinalysis parameters were established, and values compared to other published values, and in rabbits with suspected chronic kidney disease. Rabbits with suspected chronic renal disease had higher urine protein levels, higher urinary protein-to-creatinine ratios, lower urinary creatinine levels, lower urine specific gravity, and an elevated urinary gamma-glutamyl transferase index [138]. Differences were noted in this group versus purebred rabbit groups, highlighting the possibility of variation between rabbit breeds and testing modalities.

#### 5.3.2. Imaging

Modalities useful for evaluating kidneys in rabbits include radiography, ultrasound, CT imaging, and MRI, with or without contrast. Radiographic assessment of kidneys gives information on the size, shape, and position (average kidney size is 1.4–2.2 times the length of the second lumbar vertebra) [139]. Normal renal appearance via ultrasonographic evaluation has been evaluated [140]. With *E. cuniculi* infection, kidneys may be small and irregular due to chronic damage, and there is typically a loss of boundary between the cortex and medulla or cortical thinning [67].

Contrast urography (excretory urography) can help assess kidney perfusion and glomerular filtration, and detect obstructive disease and other abnormalities of the ureters [141]. Contrast is typically administered IV using dosages proposed for cats. However, one study described intraosseous administration of contrast in rabbits, which produced results identical to those when contrast was administered IV [142]. Another study utilized digital fluoroscopic excretory urography in healthy New Zealand White rabbits to determine phases of the urogram. Lopamidol was administered at 850 mg iodine/kg over 30 s. The beginning of the nephrographic phase occurred at a median time of 10 s, while the pyelographic phase began at a median time of 1.39 min. Contrast was observed in the bladder at a median time of 1.58 min [143].

The glomerular filtration rate (GFR) by means of plasma clearance of iohexol was evaluated in healthy research domestic rabbits. Iohexol (64.7 mg/kg) was administered IV and multiple blood samples obtained at six timepoints. This method proved safe and accurate in healthy research rabbits and may have applications for clinical practice [144]. Another single blood sample method to evaluate GFR was investigated in healthy laboratory rabbits using a single injection of inulin (40 mg/kg IV). In another part of the study, rabbits were given IV cisplatin, and GFR was noted to decrease before BUN and creatinine levels increased [145]. In both studies, GFR was reported as between 4.0 and 4.5 mL/min/kg.

Information on the appearance of the kidneys when using contrast computed tomography was established in 23 healthy rabbits [146]. Another retrospective study focused on the safety of IV-administered contrast medium for CT imaging; 350 rabbits were included, and there were no episodes of acute reactions or death. Eight rabbits died within the first 7 days post contrast administration and scan, and all were presumed to be due to disease sequelae and not administration of contrast. For each rabbit, a bolus of 740 mg iodine/kg of non-ionic iodinated contrast medium was injected into the marginal auricular vein [95].

Contrast-enhanced ultrasound was investigated in a reperfusion injury model in rabbits. Maximal changes occurred 3 days after injury, and this imaging modality may be useful to monitor the severity of tissue damage in cases of acute kidney damage [147].

Ante mortem kidney biopsy is feasible, and the most direct approach is likely via a flank incision (A.L., personal communication). While all modalities may be useful for evaluation of overall renal health and function, confirming if acute or chronic changes are a result of *E. cuniculi* infection is challenging [137].

### 5.4. Treatment

#### 5.4.1. Conventional Treatment

While robust studies and clinical trials are lacking in the evaluation of the treatment of renal disease in pet rabbits, the general clinical consensus is that treatment plans extrapolated from feline medicine are effective in the long-term management of renal disease in this species [148]. Conventional treatment of renal disease in rabbits with suspected or confirmed *E. cuniculi* infection requires a comprehensive approach that targets parasite control, renal support, and management of systemic complications (Table 2).

Benzimidazoles such as fenbendazole (20 mg/kg PO q24 h) and oxibendazole (15 mg/kg PO q24 h) are commonly used for 28 days for their anti-microsporidial effects (see also Section 4.4.1) [97,99]. While some clinicians use these drugs for short-term periods, extended treatment may be warranted in chronic or neurological cases, although periodic CBC monitoring is recommended due to the risk of bone marrow suppression [52,99,103,149,150].

Supportive care with subcutaneous or intravenous fluids at 2–4 mL/kg/h IV or 50–100 mL/kg/d (SC in several sites, divided q6–8 h) is crucial to correct dehydration and improve renal perfusion [110]. The fluid required to correct dehydration (in ml) is calculated from body weight (kg) × estimated dehydration (%) × 1000, and this (along with maintenance fluids) is typically provided over 24–48 h [99,148].

In cases of acute decompensation, intravenous fluids at the higher rate are preferred to restore effective circulation and prevent further tubular damage [150,151].

Dietary modifications are essential in rabbits with chronic kidney disease. These include limiting excessive calcium and phosphorus intake by emphasizing grass hay and low-calcium greens. Electrolyte imbalances and metabolic acidosis may be managed with oral bicarbonate therapy and phosphate binders when necessary (Table 2) [150,151].

**Table 2:** Common medications for renal management in rabbits [99,101].

Category	Drug	Dose	Route/Frequency	Indication
Anti-microsporidial	Fenbendazole	20 mg/kg	PO q24 h × 28 d	Microsporidial infection
	Oxibendazole	15 mg/kg	PO q24 h × 28 d	Microsporidial infection
Antihypertensive	Telmisartan	1–2 mg/kg	PO q24 h	Proteinuria, hypertension
	Benazepril	0.25–0.5 mg/kg	PO q24 h	ACE inhibitor for proteinuria
	Enalapril	0.25–0.5 mg/kg	PO q24 h	ACE inhibitor for proteinuria
	Amlodipine	0.0625 to 0.5 mg/kg	PO q24 h	Systemic hypertension *
NSAID	Meloxicam	0.5 mg/kg, up to 1 mg/kg	PO q12 h PO q24 h	Anti-inflammatory (use cautiously in renal disease and ensure good hydration)
Hydration	Subcutaneous fluids	50–100 mL/kg/d	IV or SC (in several sites, divided q6–8 h)	Rates given dependent on hydration status
Antianemic	Iron supplementation	Iron dextran 4–6 mg/kg IM q7 d; ferrous sulfate 4–6 mg/kg PO q24 h	Injectable/PO	Address CKD-associated anemia
	Aluminum hydroxide	30–60 mg/kg	PO q8–12 h	Phosphate binder
	Epoetin alfa	50–150 U/kg	SC q2–3 d	Stimulates erythropoiesis, use until PCV is normal, the q7 d for at least 4 weeks

* Dose rate is anecdotal and extrapolated from cats [152]. Key: mg = milligram, kg = kilogram, mL = milliliters, q = every, PO = per os, SC = subcutaneous, IM = intramuscular, IV = intravenous, h = hour, d = day.

To manage hypertension and reduce proteinuria, angiotensin receptor blockers such as telmisartan (1–2 mg/kg PO q24 h) are used alongside calcium channel blockers like amlodipine (0.0625–0.5 mg/kg PO q24 h extrapolated from cat dose) [152]. The use of angiotensin-converting enzyme (ACE) inhibitors, including benazepril (0.25–0.5 mg/kg PO q24 h) and enalapril (0.25–0.5 mg/kg PO q24 h), has also been extrapolated from canine and feline medicine and may be beneficial in rabbits, though published data is limited [99,149,150,153] (Table 2).

Chronic inflammation and pain may be addressed in certain rabbits with NSAIDs such as meloxicam (0.5 mg/kg PO q12 h up to 1 mg/kg PO q24 h), used cautiously in euhydrated patients with stable renal values. Studies have shown that rabbits may require a dose exceeding 0.3 mg/kg q24 h to achieve optimal plasma levels of meloxicam over a 24 h interval, and doses of 1–1.5 mg/kg SC, PO are well tolerated for 5 days [104,154,155]. Evidence suggests a 6–8 h half-life for this drug in rabbits, and so the total daily dose may be divided to give 0.5 mg/kg PO q12 h [154], (Table 2). Nephrotoxic drugs such as aminoglycosides must be avoided in compromised patients [99,150,153].

When normocytic, normochromic anemia develops because of chronic kidney disease, iron supplementation or erythropoiesis-stimulating agents may be considered, guided by hematologic monitoring (Table 2) (J.G., E.K., personal communication [99]). The use of erythropoiesis-stimulating agents is extrapolated from other species, and these therapies have not been evaluated in rabbits, except in experimental settings [156,157]. Phosphate binders, such as aluminum hydroxide, can also be used in cases of hyperphosphatemia due to renal failure (Table 2) [99].

Client education is key to long-term management. With consistent care, rabbits with renal disease may maintain a good quality of life over extended periods [111,150,151]

- Follow-up monitoring

Ongoing monitoring is essential in rabbits with renal disease to assess the treatment response, detect progression, and adjust therapeutic plans. Regular follow-up evaluations should include a combination of physical examination, laboratory testing, and blood pressure measurement if this is available [150]. Quality-of-life assessment should be regularly performed in chronic renal disease patients to ensure good animal welfare [158].

Systemic blood pressure should be monitored every 4–8 weeks in rabbits receiving antihypertensive or antiproteinuric therapy (J.G., personal communication). Persistent hypertension can contribute to further glomerular damage and exacerbate proteinuria [148,150,151].

Urinalysis should be performed regularly to evaluate urine specific gravity, sediment, and the presence of protein, glucose, or casts. The urine protein:creatinine ratio provides a quantitative measure of proteinuria; a value greater than 0.40 is suggestive of renal pathology in rabbits [138,150].

In addition, SDMA may detect renal dysfunction earlier than creatinine. Although validated primarily in cats and dogs, values above 14 µg/dL may indicate reduced renal function [151], with the normal reference interval reported as 4–18 µg/dL [136]. Further studies are needed to evaluate its use for early detection of renal disease in pet rabbits.

Routine blood work, including a CBC and serum biochemistry, should be repeated every 1–3 months depending on disease severity (J.G., E.K., personal communication [148]). Monitoring parameters include the packed cell volume (for anemia), serum creatinine, BUN, phosphorus, calcium, and electrolytes. Rising creatinine or BUN may indicate a declining renal function, while hyperphosphatemia is a concern in chronic disease [111,150].

Concurrent illnesses, which could affect the management of renal disease in rabbits, should also be considered, such as underlying cardiac disease. Body weight, body condition score, appetite, hydration status, and clinical signs such as polyuria/polydipsia, urine scald, or lethargy should be monitored at home and during each recheck exam. Adjustment of fluid support, medications, urinary scald management, and dietary plans should be based on these findings [148].

#### 5.4.2. Supportive Care and Alternative Treatments

In addition to medical treatment of renal disease, there are several supportive treatment options.

- Hydration and nutritional support

Renal disease and azotemia may be managed longer-term with diuresis via chronic subcutaneous fluid administration (S.K., personal communication). However, the benefit to longevity of chronic subcutaneous fluid therapy is still to be evaluated in rabbits with renal disease, and this needs to be weighed against the stress to both animal and owner, as well as risks involved [148]. Provision of repeated subcutaneous fluid therapy by owners at home needs to be assessed on a case-by-case basis (S.K., personal communication). Rabbits should be carefully monitored to ensure there is a clear clinical benefit, to avoid overhydration, and to monitor for signs of infection or reaction at injection sites.

Rabbit owners can increase the water intake by offering syringe feeding formula and wetting down the greens or by increasing the proportion of fresh food (S.K., personal communication). It is important to make sure the rabbit always has constant access to water. A water fountain may help to entice them to drink more. Using a ceramic bowl is recommended over a sipper bottle, as sipper bottles may block, are not easy to clean, and are associated with a lower daily water intake. Bowls allow rabbits to drink in a more natural manner [159].

If there is a protein-losing nephropathy, the rabbit may develop hypoproteinemia, leading to muscle loss and a reduced body condition. Feeding larger amounts of highly concentrated food such as pellets and syringe feeding formulas can help maintain a good weight (S.K., personal communication). Owners can purchase an electronic baby scale to monitor the weight regularly at home. If the rabbit has calcium buildup in the urinary tract, alfalfa hay and pellets are not recommended [150]. In addition, the rabbit should be encouraged to exercise regularly to aid in the suspension of crystals in the urine, which will help increase elimination via urination and reduce the buildup of sludge [150]. If the rabbit has concurrent musculoskeletal or neurological issues, it may have trouble accessing food.

- Alternative treatments

Traditional Chinese veterinary medicine (TCVM) has been used in animals for thousands of years. Acupuncture and herbal formulas may help improve renal function and slow disease progression [160,161,162,163]. If complementary treatment is desired, owners of rabbits with renal disease should consult with a TCVM-certified veterinarian, who may recommend a combination of acupuncture needling, acupressure, and herbal remedies (S.K., personal communication).

- Acupuncture

The exact mechanism for how acupuncture works is not truly known. Anecdotal evidence and several studies have shown positive results when using acupuncture to treat renal disease in humans and rats [160,161,162,164]. Acupuncture can have a neuromodulatory effect on autonomic tone and can have an antihypertensive effect. It can improve renal blood flow and GFR, decrease urinary albumin secretion, protect glomeruli and renal tubules, and thus improve renal function and slow the progression of renal disease in diabetic nephropathies in humans [163]. Acupuncture points have been described in rabbits, and its use has shown beneficial effects in experimentally induced nephritis in a rabbit model [165,166].

- Supplements

1. Herbal formulas

There are numerous Chinese herbal mixtures for treating kidney disease in animals, which could potentially be helpful in pet rabbits with renal disease, although studies in this species are lacking [162]. It is advisable to either use formulas recommended by a TCVM-certified veterinarian or use a packaged formula from a reputable company specifically labeled for treatment of renal disease in animals (S.K., personal communication). It is challenging to define and study the chemical compounds present in herbal formulas, but there have been many studies demonstrating an improvement in renal function with herbal formula dietary supplementation [162]. Chinese herbs can be renoprotective, and they can lower creatinine, increase inulin clearance, stimulate the immune system, promote diuretic activity, decrease glomerular hyperperfusion and proteinuria, and improve the plasma levels of total cholesterol and albumin [162]. Rehmannia 8 is a frequently used herbal formula that has been shown to decrease proteinuria and is renoprotective with anti-inflammatory, antioxidant, antiapoptotic, and antifibrotic properties in experimental studies in mice and human clinical trials [167,168,169]. There is some experimental evidence for its use in rabbits with renal disease; however, effects may vary with product formulation [170].

2. Antioxidants and omega-3 fatty acids

Supplements such as antioxidants and omega-3 fatty acids have been shown to be beneficial in animals with renal disease [162]. Antioxidants such as vitamin C and vitamin E protect cells from damage caused by free radicals in cats with spontaneous renal insufficiency, when fed as supplements over a 4-week period [171]. Antioxidants relax smooth muscle and increase GFR [170].

Omega-3 fatty acids can heal glomerular and interstitial lesions, and they slow the progression of renal disease [172]. They can also prevent proteinuria, decrease intrarenal calcification, and have anti-inflammatory and antioxidant effects in animals and humans [162,172]. They are most frequently sourced from fish oil. Since rabbits are herbivores, plant sources of omega-3 fatty acids should be used, such as flaxseeds and flaxseed oil (S.K., personal communication). These contain a short-chain polyunsaturated fatty acid called alpha-linolenic acid, which has been shown to decrease glomerular injury, slow the decline in renal function, and improve blood pressure in an experimental rat model with renal ablation, when incorporated as 15% of the diet [173].

3. Iron

Animals with renal disease often develop anemia due to decreased production of erythropoietin. Anemia can be treated with weekly injections of iron dextran at 4–6 mg/kg IM q7 d [99]. Rabbits may also benefit from daily oral iron supplementation (ferrous sulfate 4–6 mg/kg PO q24 h) (Table 2) [99]. Severe anemia can be treated with injections of epoetin alfa (50–150 U/kg SC q2–3 d) (Table 2) [99,148].

4. L-carnitine

L-carnitine is an amino acid that helps break down fatty acids and transport them into cells to be used for energy. It has been shown to be beneficial in reducing kidney damage in rats, due to its antioxidant effects [174], and has been shown to improve cognition in rat experimental models [175]. Research in rabbits with experimentally induced renal disease indicates that L-carnitine can have a protective role, reducing oxidative renal injury [176,177].

This suggests that dietary L-carnitine supplementation could be helpful in rabbits with chronic renal disease, but further research is necessary to determine its efficacy in this species. It has also been associated with an increased skeletal muscle mass in a 20-day experimental feeding trial in piglets, and thus it could also potentially be beneficial in rabbits with muscle wasting secondary to protein losing nephropathy [178]. Further scientific studies are needed to evaluate dose rates and the efficacy of using these supplements in pet rabbits with renal disease, as well as to determine any potential side effects.

## 6. Ocular Presentation

### 6.1. Clinical Overview and Epidemiology

**Figure 8:**
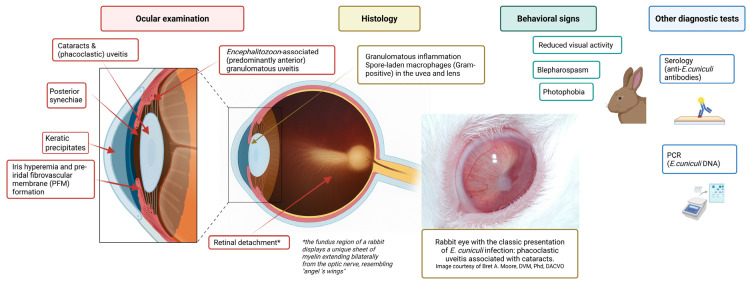
*Encephalitozoon cuniculi* can cause various ocular alterations detectable during ophthalmological examination or after enucleation. Behavioral changes due to impaired vision and ocular disease may necessitate phacoclastic emulsification of the affected eye lens, and spores can be detected by PCR in removed tissue. The photograph of the eye shows a close-up view of the left eye of a rabbit with the classic presentation of ocular infection caused by *E. cuniculi*: phacoclastic uveitis (resulting from the rupture of the lens capsule) associated with cataracts. Note the whitish abscess in the anterior uvea. Figure created in https://BioRender.com.

Ocular signs are clinically significant in rabbits, with studies reporting that up to 20–40% of seropositive rabbits develop ocular manifestations such as uveitis or cataracts [33,34]. Common ocular lesions in infected rabbits include chronic anterior uveitis and cataract formation, with rupture of the lens capsule leading to phacoclastic uveitis (Figure 8). Several studies on pet rabbit populations have confirmed a generally higher prevalence of seropositivity within subpopulations of rabbits displaying active clinical signs consistent with encephalitozoonosis, including cases with cataracts, uveitis, or other ocular signs [36,179,180], while one study showed a higher seropositivity in asymptomatic rabbits [70].

Similarly, ocular lesions—including focal cortical anterior cataracts associated with anterior uveitis—have been increasingly documented in cats and dogs with *E. cuniculi*, and isolated cases in exotic felids such as snow leopards have also been reported [181,182]. However, ocular *E. cuniculi* remains underrecognized largely due to the lack of routine diagnostic testing (such as PCR on aqueous humor or lens material), the nonspecific nature of ocular lesions that are often attributed to more common causes of uveitis or cataracts, and limited awareness among veterinarians regarding its significance as a differential diagnosis in small, exotic, and wild animals [21,40,88]. Notably, greater awareness and implementation of better diagnostic methods are demonstrating that *E. cuniculi* can also contribute to a wide range of eye lesions in HIV-infected and non-HIV-infected humans [183].

**Figure 9:**
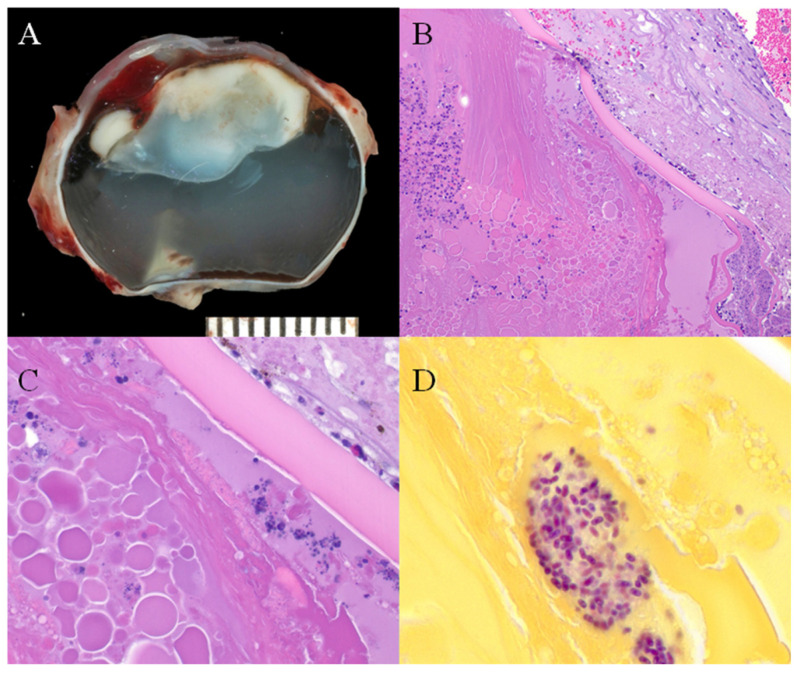
Formalin-fixed globe from a 1-year-old mini lop rabbit. (**A**). Gross image of the hemisected globe displaying hyphema, cataracts, and granulomatous inflammation of the lens and iris. (**B**). Photomicrograph showing the frayed and discontinuous lens capsule, lens fiber degeneration, inflammatory cell infiltration (phakitis) and overlaying fibrovascular membrane (**C**). Higher magnification image of lens showing lens fiber liquefaction, Morgagnian globule formation, and inflammatory cells and debris inside the lens. (D). High magnification image of lens showing basophilic organisms contained within a macrophage (Gram stain). Courtesy of Gillian C. Shaw, Comparative Ocular Pathology Laboratory of Wisconsin (COPLOW), University of Wisconsin-Madison.

Histopathological examination of affected ocular tissues reveals granulomatous inflammation, lens capsule rupture, and spore-laden macrophages within uveal tissues (Figure 9) [184]. In rabbits, lesions often include fibrovascular membrane formation (Figure 9), severe lens-induced uveitis, and even retinal detachment. In dogs and other mammals, ocular histopathology may similarly demonstrate granulomatous uveitis with spores within affected tissues. Spores can also be sporadically found in the retina, causing significant focal degeneration [82]. The presence of spores, identifiable with specific staining techniques, particularly the modified trichrome stain (MTS) and Gram stain, visualized using light microscopy, as well as calcofluor white stain, observed under ultraviolet light microscopy, is the most effective method for detecting *E. cuniculi* spores in paraffin-embedded tissues (Figure 9). These staining methods have shown greater efficacy compared to Warthin–Starry, Ziehl–Neelsen, Giemsa, and periodic acid–Schiff (PAS) reactions for identifying spores with minimal background ‘noise’ or monochromatic interference. Furthermore, these stains allow for the clear identification of individual spores within paraffin-embedded tissues. PCR is a highly sensitive diagnostic tool for detecting *E. cuniculi* DNA in ocular samples, including aqueous humor or lens material [185]. This method is critical for confirming active infection and distinguishing *E. cuniculi* from other potential causes of uveitis [185].

### 6.2. Clinical Signalment

**Figure 10:**
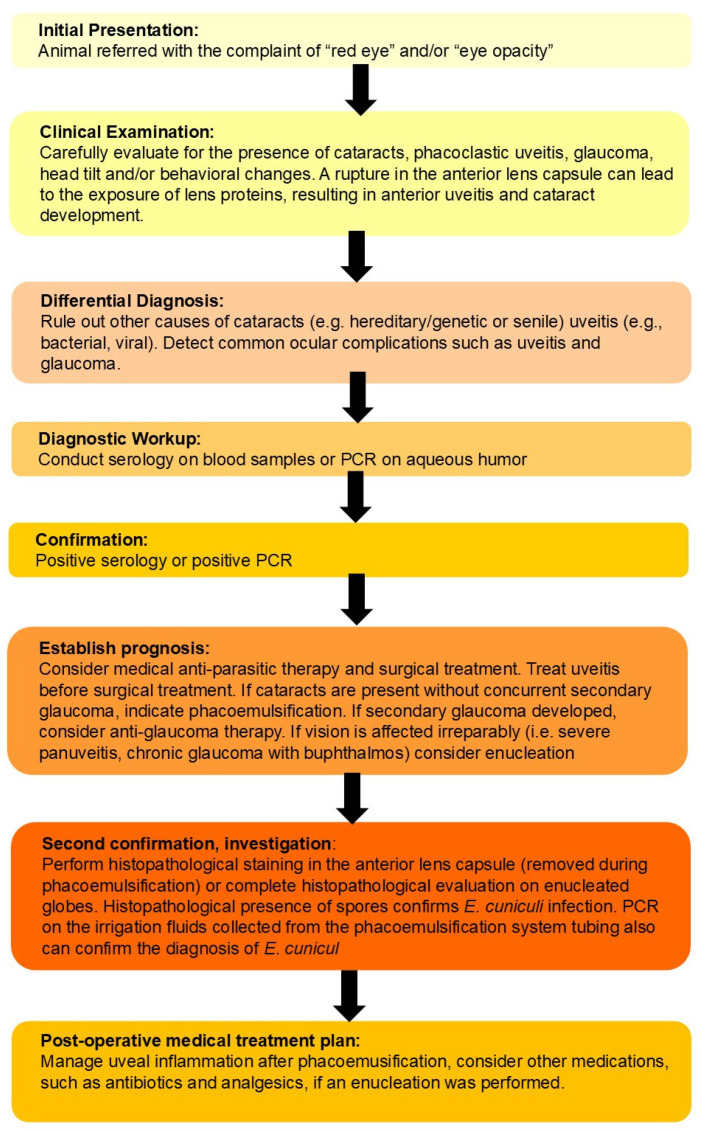
Flow chart for ocular infection caused by *Encephalitozoon cuniculi* in rabbits.

The clinical examination of animals suspected of ocular infection due to *E. cuniculi* should include a thorough inspection of the ocular adnexa, as well as the anterior and posterior eye segments (Figure 10, Table 3) [36,186]. Typical ocular signs in rabbits include phacoclastic uveitis, usually unilateral, with associated inflammation and cataracts, presenting as white, ruptured lenses [36,186]. *E. cuniculi* shows a particular affinity for the lens, which may be a more common initial infection site in rabbits acquiring the organism through vertical transmission [137,187]. In fact, in a recent survey among veterinarians, ocular disease was indeed more frequently observed in younger rabbits and was reported to be commonly presented as a combination of uveitis and cataracts, in about 88.2% of cases. Uveitis alone was observed in 31.8% of cases, and cataracts alone in 22.4% [40]. Once the lens is infected, as confirmed immunohistochemically [188], parasitophorous vacuoles may rupture, leading to an acute lens capsule rupture and a severe granulomatous phacoclastic uveitis, mainly affecting the anterior uvea (the iris and ciliary body) [88]. Alongside key clinical signs of uveitis, such as aqueous flare, miosis, and iris vessel congestion (rubeosis iridis), *E. cuniculi*-induced uveitis often presents as white-to-pale pink nodular granulomas in the iris (Figure 8). Infection by *E. cuniculi* is the leading and most frequent cause of uveitis in rabbits. Ocular changes can progress to glaucoma or phthisis bulbi if untreated. Secondary glaucoma frequently occurs in rabbits with ocular lesions caused by *E. cuniculi* [189]. Additional findings during an ophthalmological examination may include corneal edema, epithelial ulceration, endothelial necrosis, keratic precipitates, rubeosis iridis, and posterior synechiae [82,184,190]. Besides uveal and lenticular lesions, *E. cuniculi* can potentially cause other, less common ocular manifestations. The organism has been detected using immunohistochemistry in several ocular structures in immunocompetent rabbits after experimental oral infection, including the periocular connective tissue, sclera, cornea, and retina [36]. Other signs, such as photophobia, blepharospasm, and reduced visual acuity, should also be investigated [184,187,190]. In dogs and other mammals, similar signs of uveitis and cataract formation may occur, though the frequency and severity can vary depending on the species and immune response [191].

### 6.3. Treatment

Treating ocular infections caused by *E. cuniculi* requires a combination of anti-microsporidial and anti-inflammatory medications, along with surgical options (Figure 10) [186]. Regardless of the underlying cause, anterior uveitis may be empirically managed with ophthalmic corticosteroids, in the absence of corneal ulceration. When corneal ulcers are present, an ophthalmic NSAID is indicated. Systemic NSAIDs, such as meloxicam, have proven effective in managing chronic uveitis associated with *E. cuniculi,* and long-term NSAID use may be necessary [34,52,88]. Although the administration of systemic corticosteroids in rabbits with encephalitozoonosis is generally discouraged because of documented immunosuppression and a lack of proven benefit, topical ophthalmic corticosteroids (e.g., prednisolone acetate 1% eye drops) may be used to control anterior uveitis in *E. cuniculi*-associated phacoclastic uveitis, especially following phacoemulsification, and in combination with anti-microsporidial therapy [21,96,105,188,192].

However, the primary approach is to target the specific cause of uveitis, if identifiable. In rabbits affected by encephalitozoonosis, treating the infection with a benzimidazole (e.g., fenbendazole, or oxibendazole) is commonly used to target the parasite, administered over a 28-day course [186,187] (Table 1 and Table 2). Unfortunately, many rabbits with uveitis linked to *E. cuniculi* do not respond well to medical treatments [193], and painful or vision-impairing complications may arise, such as secondary glaucoma and progressive cataracts. In cases where the globe is blinded and painful, enucleation or chemical ablation is often performed for presumptive relief [189]. Uveitis associated with *E. cuniculi* is frequently challenging to manage with medication alone, and secondary cataract formation cannot be reversed through medical treatment. Because in the presence of cataracts, the uveal inflammation is mainly lens-induced, treatment often involves lens removal via phacoemulsification, combined with topical anti-inflammatory medication post-operatively to restore vision and reduce the surgically induced intraocular inflammation [192,194,195,196]. This approach aims to control the local ocular infection, as supported by published case reports and small case series showing successful phacoemulsification in domestic rabbits [192,193,194,195,197]. The best clinical visual outcome after surgery usually occurs in patients who undergo phacoemulsification in the early stages of cataract development (incipient and immature stages).

In cases where phacoemulsification is used as part of the treatment, irrigation fluids collected from the tubing of the instruments were sent for PCR, confirming the diagnosis of *E. cuniculi* [192]. Artificial intraocular lens (IOL) implantation may be performed postoperatively; one study described successful size and dioptric power calculations for rabbit IOLs, and another case report documented bilateral IOL placement in a pet rabbit with a favorable outcome [196,198]. However, IOL placement is not always indicated—especially in the presence of ongoing inflammation, a small ocular anatomy, or surgical complications.

Moreover, rabbits exhibit significant lens fiber re-growth and posterior capsular opacification; this regenerative trait can make IOL outcomes less predictable and, in some cases, supports the decision to leave the eye aphakic (without an IOL) [199]. Most rabbits that are left aphakic can cope well visually over time, thanks to their naturally wide visual field and strong reliance on periorbital senses; quality of life can remain good even without an artificial lens.

Evaluation of the serostatus can have limited diagnostic value given the high seroprevalence in rabbits. However, when a typical ocular lesion (such as phacoclastic uveitis) occurs alongside a positive *E. cuniculi* serology, the diagnosis can be regarded as probable infection rather than merely a tentative diagnosis. Definitive confirmation would require further workup including a histopathological demonstration of the organism after enucleation (a procedure reserved for painful, non-visual eyes) or PCR of liquefied lens material after phacoemulsification [192]. In clinical settings, however, cases with both characteristic lesions and positive serology are treated as probable *E. cuniculi* infections [192].

## 7. Client Communication on Prevention of Zoonotic Transmission

As an opportunistic human pathogen, *E. cuniculi* can affect specifically immunocompromised patients (see Section 1. Introduction). If the animal owners or those who are handling the infected rabbits are concerned about the possibility of adverse effects of an infection of the animals and their own and their family’s or coworkers’ health, this risk should be evaluated on an individual basis with a focus on possibly immunocompromised contact persons (e.g., hematopoietic stem cell and transplant recipients, chemotherapy patients, and others) and adequate personal hygiene, such as frequent hand washing and minimizing contact with animal excreta. Veterinarians play a primary role in zoonosis transmission, especially in such specific cases [200,201].

## 8. Summary Statements

- *E. cuniculi* is a microsporidian parasite affecting various mammals, with notable infection in rabbits. In principle, the four genotypes have a different species predilection, but all of them have been described to infect humans.
- The excretion of infectious spores in large numbers by domestic and wild hosts leads to permanent environmental contamination and a subsequent exposure of new hosts.
- Upon infection of rabbits, *E. cuniculi* quickly spreads into various organs, multiplying, disseminating and finally inducing chronic infections in the kidney and brain as primary target organs.
- Experimental infection with *E. cuniculi* in adult, immunocompetent rabbits predominantly results in a subclinical infection, with the activation of both humoral and cell-mediated immune responses.
- Cell-mediated immunity is characterized by the ability of both CD4+ and CD8+ T cells to proliferate after stimulation with specific antigens.
- TH1 polarization of the immune response with predominant IFN-γ expression can be detected in the spleen, mesenteric lymph nodes, and Peyer’s patches.
- The elevated expression of IL-4 and IL-10 mRNA in the small intestine could be indicative of a balanced control of IFN-γ that prevents tissue damage and allows the organism to persist.
- In rabbits, *E. cuniculi* occurs worldwide, with seroprevalence rates of up to 85%.
- The various available serological methods correlate but do not produce equivalent results.
- In an immunocompetent rabbit, a negative serological result should be considered confirmatory of non-infection unless exposure has been very recent.
- Pathohistological lesions in predilection organs (CNS and kidney) are not significantly different in rabbits with and without clinical signs, and the determination of the grade of pathomorphological lesions alone cannot be considered a reliable post mortem diagnostic tool to confirm *E. cuniculi* as a cause of clinical disease.
- Neurological signs associated with *E. cuniculi* infection almost always show only signs of peripheral vestibular disease instead of central vestibular disorders.
- The main differentials for vestibular dysfunction in rabbits include encephalitozoonosis and otitis media/interna. Facial nerve spasticity is a clear indication of otitis media/interna and not of encephalitozoonosis. It should be noted that these diseases can occur concurrently.
- Therapeutic exercise may represent the most important part of treatment in rabbits with vestibular disease.
- A prolonged period of immobilization because of acute vestibular disorder may limit the recovery of vestibular dysfunction.
- For inhibition of spore proliferation, benzimidazole preparations (e.g., fenbendazole) have been shown to be effective.
- The kidney is a predilection site for the microsporidian parasite *E. cuniculi,* resulting in chronic interstitial nephritis.
- Most cases of renal infection remain clinically latent, only resulting in renal failure in the advanced stage of infection.
- Clinical signs of renal failure can be non-specific in pet rabbits, but may include lethargy, depression, anorexia, teeth grinding, weight loss, reduced body condition, anemia, polyuria, polydipsia, and perineal urine scald.
- Diagnostic workup for kidney disease is similar to that in other species, including bloodwork, urinalysis, and imaging modalities such as radiography, ultrasound, CT, and MRI, with or without contrast.
- Conventional treatment of renal disease in rabbits with suspected or confirmed *E. cuniculi* infection requires a comprehensive approach that targets parasite control, renal support, and management of systemic complications.
- Alternative treatments for renal disease may be considered, including acupuncture and supplements such as herbal formulas, antioxidants, omega-3-fatty acids, iron, and L-carnitine.
- Client education is key to long-term management of renal disease in pet rabbits.
- Physical examination findings in rabbits with ocular infection often reveal phacoclastic uveitis (ruptured lenses) and potential secondary complications such as glaucoma.
- Histopathology shows granulomatous inflammation of the uvea and spore presence, especially inside the lens tissue, where there is a predilection for the parasite.
- Treatment of ocular infection involves anti-microsporidial drugs (e.g., fenbendazole), anti-inflammatory management for the uveitis, and cataract surgery (phacoemulsification).
- For immunocompromised people that are in contact with rabbits, the specific risks of zoonotic transmission should be considered and communicated to the owner together with preventative measures.

## 9. Challenges and Future Directions

### 9.1. Atypical Case Presentation: Gastrointestinal Manifestations of E. cuniculi

While neurological, renal, and ocular signs are the most classically described manifestations of *E. cuniculi* infection in rabbits, increasing anecdotal clinical observations suggest that recurrent episodes of gastrointestinal hypomotility, often labeled as rabbit gastrointestinal syndrome, may represent an atypical but significant presentation.

In some patients, gastrointestinal signs can progress over time and may be eventually associated with mild neurological or renal abnormalities, such as rear limb ataxia, urine dribbling leading to urine staining of the perineum, or intermittent azotemia. Neurological dysfunction affecting autonomic innervation to the GI tract has been proposed as a possible mechanism for this presentation. Potential differential diagnoses for these clinical signs should also be ruled out through use of appropriate diagnostic testing.

In one case example, a rabbit with a multi-year history of intermittent RGIS episodes showed progressive azotemia, elevated inflammatory markers, and strong *E. cuniculi* seropositivity (J.G., personal communication). Necropsy findings in a second case revealed classic lesions of microsporidiosis in the kidney and liver, as well as suspected neurogenic functional ileus secondary to encephalitozoonosis (J.G., personal communication). This suggests that in some rabbits, recurring GI stasis may reflect low-grade, chronic neurological dysfunction related to *E. cuniculi*-induced inflammation of the enteric nervous system or spinal cord.

A retrospective study indicated that approximately 27% of seropositive rabbits exhibited GI signs such as chronic weight loss, anorexia, and recurrent GI hypomotility episodes, suggesting a potential link between *E. cuniculi* and GI dysfunction [67]. In a recent international survey of exotic and small animal veterinarians, approximately half of the respondents, U.S. 54.0% (88/163) and non-U.S. 51.1% (90/176), stated that GI stasis could be observed with *E. cuniculi* infection in the absence of other clinical signs normally associated with infection (i.e., neurological, ocular, renal) [40].

Though controlled studies are lacking, clinical experience supports the inclusion of *E. cuniculi* in the differential diagnosis for idiopathic or relapsing RGIS, particularly in patients with concurrent neurological or urinary abnormalities. Continued investigation into the GI manifestations of this pathogen is warranted.

### 9.2. Future Directions: Developments of Anti-Microsporidial Treatments

Current recommendation for the etiological treatment of rabbit encephalitozoonosis include the benzimidazoles fenbendazole and oxibendazole. These limited options warrant further research on anti-microsporidial compounds that can be applied as therapeutics or preventatives. This includes the antirheumatic (anti-inflammatory) auranofin (which has been shown to be effective against *E. intestinalis* in mice [202]), antimycotic drugs such as fumagillin (a mycotoxin with a broad-spectrum efficacy against various protozoa, registered for treatment of microsporidiosis in humans; [203]), as well as a number of compounds that have shown to be effective in vitro, such as different polyamines, lipase blockers, chitin synthase inhibitors, calcium antagonists, and calmodulin inhibitors, as well as plant extracts from wormwood (*Artemisia*) or laurel [204]. For registration or repurposing, experimental and field studies will be required to demonstrate efficacy as well as safety, and the pharmaceutical industry should be made aware of the needs for such intervention measures in veterinary (pet) medicine.

### 9.3. A Plan to Better Define and Understand E. cuniculi Infection

#### 9.3.1. Towards Better Diagnostics for Neurological Presentations

More research is needed to understand how pathological changes observed with *E. cuniculi* infection of the brain and spinal cord correlate clinically in pet rabbits. To improve the in vivo diagnosis of the neurological form of encephalitozoonosis, further comprehensive studies are needed to identify and to classify underlying etiologies for meningoencephalitis other than *E. cuniculi* infection. Then, from this information, new methods should be developed to facilitate ruling out relevant differential diagnoses.

#### 9.3.2. Better Understanding of Renal Complications of Infection

It is generally acknowledged that *E. cuniculi* infection is a cause of renal disease in rabbits as they age. Epidemiological studies should be conducted on naturally infected rabbits with severe renal disease to determine the presence of *E. cuniculi* and *E. cuniculi*-related renal changes at the histological level. Based on this information, it may be proposed that rabbits that are seropositive, and especially those that have previously been treated for neurological clinical signs, should be monitored more closely via blood chemistry testing to better gauge the onset of subclinical renal disease, whereby supportive therapies may be initiated.

#### 9.3.3. Gauging the Prevalence of Ocular Infection

Studies of naturally infected rabbits have defined vertical transmission as the likely origin of ocular infection, although authors reporting an experimental model using oral inoculation also noted this route as a possible mode of infection. Further research should be conducted through donated eye tissue for histopathology and molecular diagnostic testing regardless of the case presentation to understand the prevalence of this predilection site of infection in pet rabbits.

#### 9.3.4. Definition of the Pathogenesis of Active and Chronic/Relapsing Infection

Active infection should be defined as the first instance of presentation of clinical signs. It is hypothesized that chronic/relapsing infection may occur via three pathways. First, through reactivation of original infection relative to stress, underlying disease, or immune compromise. Second, through re-exposure to *E. cuniculi*, for example, in a multi-rabbit household. Third, an imbalance of existing neuropathology may arise, leading to an immune-mediated process whereby inflammation may result in the recurrence of clinical signs. To better understand these pathways, improved diagnostics are needed to detect the organism, in addition to improved assays to gauge the immune and inflammatory responses.

#### 9.3.5. Examine the Role of Immunogenetics in the Response to and Outcome of Infection

Immunogenetics is known to play a key role in the recognition and response to infectious agents. This proposal is two-fold. Based on existing information generated in laboratory rabbits, methods to determine the major histocompatibility complex and toll-like receptors should be implemented in laboratories with access to samples from pet rabbits to best understand the immunodiversity in the pet rabbit population [205,206,207]. Second, this information can be applied in tangent to current *E. cuniculi* diagnostic procedures in conjunction with obtaining information regarding case presentation and outcome.

#### 9.3.6. Examination of Immunotherapy Options in the Treatment of Infection

Long-term or latent infection with *E. cuniculi* has been proposed to be a function of an imbalance in T helper cell (Th1 and Th2) responses. Additionally, an overactive immune or inflammatory process may contribute to the neurological and renal tissue damage. In this proposal, bench-based assays to examine the immune response could be undertaken to identify animals with the Th cell imbalance. Therapies such as IFN-gamma and other agents may be considered to improve long-term outcomes. In other infections and disease processes, acute-phase proteins such as C-reactive protein and serum amyloid A have been defined as biomarkers of infection but also as mediators of tissue damage. If this type of pathology could be proven in rabbits, prototype interventions to help reign in uncontrolled inflammatory processes have been designed and would be likely preferential rather than use of wide-acting anti-inflammatory treatments.

#### 9.3.7. Preventative Measures and Treatment Options

As a measure to control the spread of *E. cuniculi* in the rabbit population, animals should be serologically tested before they are used for breeding, whereby seropositive animals should be excluded from breeding activities. In a related measure, leading pet stores should engage commercial breeders that only maintain seronegative animals.

#### 9.3.8. Drug Therapies

Due to the different manifestations of infection, a uniform treatment regimen does not exist. To date, no drugs have been approved for the causative treatment of *E. cuniculi* infection in rabbits. Future research should be focused on the treatment of natural *E. cuniculi* infections to develop a licensed drug for causal therapy of the microsporidian pathogen.

#### 9.3.9. Supportive Therapies

Recently, supportive treatment options such as physiotherapy have become more important in rabbits with vestibular dysfunction. Clinical experience in such animals indicates that treatment aimed at improving and regaining mobility seems to have a significant impact, comparable to physiotherapy for rehabilitation following strokes in humans. As physiotherapy has not yet been scientifically evaluated in rabbits displaying vestibular signs associated with *E. cuniculi* infection, studies for the assessment and further development of other supportive treatment possibilities such as this are needed.

#### 9.3.10. Defining the Risk of Zoonotic Infection

The close coexistence and the strong emotional relationship between companion animals and their owners pose a risk of zoonotic infection. Microsporidia have been frequently found to be opportunistic pathogens not only in humans with HIV infection but also in immunocompromised transplant recipients. It must be assumed that all immunocompromised humans are at risk. As *E. cuniculi* has increasingly often been associated with clinical disease in cats and dogs in recent years and has repeatedly been detected in wild immunocompetent rodents, it can be proposed that many other wild and domesticated animals pose a significant zoonotic risk for humans. Given the high rate of clinically covert infections and variability in disease presentation, infections may not be recognized in many species. In both humans and animal species, epidemiological investigations should be undertaken and include the identification of different *Encephalitozoon* spp., genotyping of isolates, the determination of prevalence, as well as pathways of pathogen transmission.

## 10. Concluding Statement

The diagnosis and treatment of *E. cuniculi* infection in rabbits remains a challenge, and it is hoped that this consensus provides guidance to both clinicians and researchers. Although experimental models of *E. cuniculi* infection have been designed, they are often unable to reproduce the clinical signs observed in pet rabbits. Thus, it is likely that most significant gains in understanding of this infection will be made through the study of patients rather than laboratory animals.

## Figures and Tables

**Table 3:** Ophthalmic clinical criteria for diagnosis of *E. cuniculi* eye infection.

Ocular Anatomic Location or Criteria	Clinical, Laboratory, or Histopathological Findings
Anterior Segment	Cataracts and uveitis (phacoclastic), keratic precipitates, iris hyperemia, posterior synechiae
Posterior Segment	Retinal detachment, granulomatous uveitis
Behavioral Signs	Photophobia, blepharospasm, reduced visual acuity
Histopathology	Granulomatous inflammation, spore-laden macrophages (positive Gram stain) in the uvea and lens
Other Diagnostic Tests	Serology, PCR for *E. cuniculi* DNA from ocular structures

## Data Availability

For this article, no new data were generated. All the relevant research papers, reports, and datasets used in the compilation of this review can be accessed through major academic databases or URLs as cited. For further clarification or additional data related to the topics covered in this review, the readers are encouraged to contact the corresponding author.

## Supplementary Materials

Four videos are available to aid in the management of rabbits with the neurological presentation of *E. cuniculi*. Video S1: Housing for the neurological rabbit (https://www.youtube.com/watch?v=JMsBpv_YGSQ, accessed on 17 January 2025); Video S2: Medicating the neurological rabbit (https://www.youtube.com/watch?v=WpPrROALmNc, access on 17 January 2025); Video S3: Handling the neurological rabbit (https://www.youtube.com/watch?v=T6UKuwVr23A, accessed on 17 January 2025); Video S4: Physiotherapy for the neurological rabbit (https://www.youtube.com/watch?v=z76nAZDYMrU, accessed on 17 January 2025).

## References

[1] Didier E.S., Weiss L.M. (2006). Microsporidiosis: Current status. Curr. Opin. Infect. Dis..

[2] Mathis A., Weber R., Deplazes P. (2005). Zoonotic potential of the microsporidia. Clin. Microbiol. Rev..

[3] NIAID Biodefense Pathogens. https://www.niaid.nih.gov/research/niaid-biodefense-pathogens.

[4] Wasson K., Peper R.L. (2000). Mammalian microsporidiosis. Vet. Pathol..

[5] Didier E., Stovall M., Green L., Brindley P., Sestak K., Didier P. (2004). Epidemiology of microsporidiosis: Sources and modes of transmission. Vet. Parasitol..

[6] Sak B., Fibigerová M., Mravcová K., Holubová N., Šikutová S., Fenclová J., Kváč M., Rudolf I. (2024). Tick-borne microsporidiosis: Ticks as a neglected source of human microsporidian infections?. Emerg. Microbes Infect..

[7] Weiss L.M., Vossbrinck C.R., Wittner M., Weiss L.M. (1999). Molecular biology, molecular phylogeny, and molecular diagnostic approaches to the microsporidia. The Microsporidia and Microsporidiosis.

[8] Ruan Y., Xu X., He Q., Li L., Guo J., Bao J., Pan G., Li T., Zhou Z. (2021). The largest meta-analysis on the global prevalence of microsporidia in mammals, avian and water provides insights into the epidemic features of these ubiquitous pathogens. Parasites Vectors.

[9] Hinney B., Sak B., Joachim A., Kváč M. (2016). More than a rabbit’s tale—*Encephalitozoon* spp. in wild mammals and birds. Int. J. Parasitol. Parasites Wildl..

[10] Anane S., Attouchi H. (2010). Microsporidiosis: Epidemiology, clinical data and therapy. Gastroenterol. Clin. Biol..

[11] Weber R., Bryan R.T. (1994). Microsporidial infections in immunodeficient and immunocompetent patients. Clin. Infect. Dis..

[12] Gumbo T., Hobbs R.E., Carlyn C., Hall G., Isada C.M. (1999). Microsporidia infection in transplant patients. Transplantation.

[13] Taghipour A., Bahadory S., Abdoli A. (2022). A systematic review and meta-analysis on the global prevalence of cattle microsporidiosis with focus on *Enterocytozoon bieneusi*: An emerging zoonotic pathogen. Prev. Vet. Med..

[14] Taghipour A., Bahadory S., Abdoli A., Javanmard E. (2022). A systematic review and meta-analysis on the global molecular epidemiology of microsporidia infection among rodents: A serious threat to public health. Acta Parasitol..

[15] Taghipour A., Olfatifar M., Foroutan M., Bahadory S., Malih N., Norouzi M. (2020). Global prevalence of *Cryptosporidium* infection in rodents: A systematic review and meta-analysis. Prev. Vet. Med..

[16] Pombert J.-F., Xu J., Smith D.R., Heiman D., Young S., Cuomo C.A., Weiss L.M., Keeling P.J. (2013). Complete genome sequences from three genetically distinct strains reveal high intraspecies genetic diversity in the microsporidian *Encephalitozoon cuniculi*. Eukaryot. Cell.

[17] Pelin A., Moteshareie H., Sak B., Selman M., Naor A., Eyahpaise M.-È., Farinelli L., Golshani A., Kvac M., Corradi N. (2016). The genome of an *Encephalitozoon cuniculi* type III strain reveals insights into the genetic diversity and mode of reproduction of a ubiquitous vertebrate pathogen. Heredity.

[18] Sak B., Brady D., Pelikánová M., Květoňová D., Rost M., Kostka M., Tolarová V., Hůzová Z., Kváč M. (2011). Unapparent microsporidial infection among immunocompetent humans in the Czech Republic. J. Clin. Microbiol..

[19] Abdoli A., Olfatifar M., Zaki L., Asghari A., Hatam-Nahavandi K., Nowak O., Pirestani M., Diaz D., Cherati M.G., Eslahi A.V. (2024). The global prevalence of microsporidia infection in rabbits as a neglected public health concern: A systematic review and meta-analysis. Prev. Vet. Med..

[20] Breuninger K., Rinder M., Korbel R. (2024). Occurrence of *Encephalitozoon cuniculi* and *Encephalitozoon hellem* in European Wild Rabbits (*Oryctolagus cuniculus*) in Southern Germany (Bavaria). Animals.

[21] Latney L.V., Bradley C.W., Wyre N.R. (2014). *Encephalitozoon cuniculi* in pet rabbits: Diagnosis and optimal management. Vet. Med..

[22] Magalhães T.R., Pinto F.F., Queiroga F.L. (2022). A multidisciplinary review about *Encephalitozoon cuniculi* in a One Health perspective. Parasitol. Res..

[23] Perec-Matysiak A., Leśniańska K., Buńkowska-Gawlik K., Čondlová Š., Sak B., Kváč M., Rajský D., Hildebrand J. (2019). The opportunistic pathogen *Encephalitozoon cuniculi* in wild living Murinae and Arvicolinae in Central Europe. Eur. J. Protistol..

[24] Němejc K., Sak B., Květoňová D., Hanzal V., Janiszewski P., Forejtek P., Rajský D., Kotková M., Ravaszová P., McEvoy J. (2014). Prevalence and diversity of *Encephalitozoon* spp. and *Enterocytozoon bieneusi* in wild boars (*Sus scrofa*) in Central Europe. Parasitol. Res..

[25] Wagnerova P., Sak B., Kvetonova D., Bunatova Z., Civisova H., Marsalek M., Kvac M. (2012). *Enterocytozoon bieneusi* and *Encephalitozoon cuniculi* in horses kept under different management systems in the Czech Republic. Vet. Parasitol..

[26] Reetz J., Nöckler K., Reckinger S., Vargas M.M., Weiske W., Broglia A. (2009). Identification of *Encephalitozoon cuniculi* genotype III and two novel genotypes of *Enterocytozoon bieneusi* in swine. Parasitol. Int..

[27] Kicia M., Zajączkowska Ż., Kváč M., Cebulski K., Holubová N., Wencel P., Mayer L., Wesołowska M., Sak B. (2022). *Encephalitozoon cuniculi* and extraintestinal microsporidiosis in bird owners. Emerg. Infect. Dis..

[28] Sak B., Vecková T., Brdíčková K., Smetana P., Hlásková L., Kicia M., Holubová N., McEvoy J., Kváč M. (2019). Experimental *Encephalitozoon cuniculi* infection acquired from fermented meat products. Foodborne Pathog. Dis..

[29] Santaniello A., Cimmino I., Dipineto L., Agognon A.L., Beguinot F., Formisano P., Fioretti A., Menna L.F., Oriente F. (2021). Zoonotic risk of *Encephalitozoon cuniculi* in animal-assisted interventions: Laboratory strategies for the diagnosis of infections in humans and animals. Int. J. Environ. Res. Public Health.

[30] Deplazes P., Mathis A., Baumgartner R., Tanner I., Weber R. (1996). Immunologic and molecular characteristics of *Encephalitozoon*-like microsporidia isolated from humans and rabbits indicate that *Encephalitozoon cuniculi* is a zoonotic parasite. Clin. Infect. Dis..

[31] Li X., Palmer R., Trout J., Fayer R. (2003). Infectivity of microsporidia spores stored in water at environmental temperatures. J. Parasitol..

[32] Koudela B., Kučerová Š., Hudcovic T. (1999). Effect of low and high temperatures on infectivity of *Encephalitozoon cuniculi* spores suspended in water. Folia Parasitol..

[33] Csokai J., Gruber A., Kunzel F., Tichy A., Joachim A. (2009). Encephalitozoonosis in pet rabbits (*Oryctolagus cuniculus*): Pathohistological findings in animals with latent infection versus clinical manifestation. Parasitol. Res..

[34] Künzel F., Gruber A., Tichy A., Edelhofer R., Nell B., Hassan J., Leschnik M., Thalhammer J.G., Joachim A. (2008). Clinical symptoms and diagnosis of encephalitozoonosis in pet rabbits. Vet. Parasitol..

[35] Hunt R.D., King N.W., Foster H.L. (1972). Encephalitozoonosis: Evidence for vertical transmission. J. Infect. Dis..

[36] Jeklová E., Levá L., Kummer V., Jekl V., Faldyna M. (2019). Immunohistochemical detection of *Encephalitozoon cuniculi* in ocular structures of immunocompetent rabbits. Animals.

[37] Cox J.C., Hamilton R.C., Attwood H.D. (1979). An investigation of the route and progression of *Encephalitozoon cuniculi* infection in adult rabbits. J. Protozool..

[38] Jeklova E., Leva L., Matiasovic J., Ondrackova P., Kummer V., Faldyna M. (2020). Characterization of humoral and cell-mediated immunity in rabbits orally infected with *Encephalitozoon cuniculi*. Vet. Res..

[39] Maestrini G., Ricci E., Cantile C., Mannella R., Mancianti F., Paci G., D’Ascenzi C., Perrucci S. (2017). *Encephalitozoon cuniculi* in rabbits: Serological screening and histopathological findings. Comp. Immunol. Microbiol. Infect. Dis..

[40] Montiani-Ferreira F., Joachim A., Künzel F., Mello F.R., Keeble E., Graham J., Martorell J., Quinton J.-F., Gottenger A., Cray C. (2024). *Encephalitozoon cuniculi* infection in rabbits (*Oryctolagus cuniculus*): Data from an international survey of exotic and small animal veterinarians. Animals.

[41] Horváth M., Švický E., Ševčíková Z. (1998). Pathomorphological response to *Encephalitozoon cuniculi* infection in cyclophosphamide-treated rabbits. Acta Vet. Brno.

[42] Jeklova E., Leva L., Kovarcik K., Matiasovic J., Kummer V., Maskova J., Skoric M., Faldyna M. (2010). Experimental oral and ocular *Encephalitozoon cuniculi* infection in rabbits. Parasitology.

[43] Jeklova E., Jekl V., Kovarcik K., Hauptman K., Koudela B., Neumayerova H., Knotek Z., Faldyna M. (2010). Usefulness of detection of specific IgM and IgG antibodies for diagnosis of clinical encephalitozoonosis in pet rabbits. Vet. Parasitol..

[44] Schmidt E.C., Shadduck J.A. (1983). Murine encephalitozoonosis model for studying the host-parasite relationship of a chronic infection. Infect. Immun..

[45] Sak B., Salát J., Horká H., Saková K., Ditrich O. (2006). Antibodies enhance the protective effect of CD4+ T lymphocytes in SCID mice perorally infected with *Encephalitozoon cuniculi*. Parasite Immunol..

[46] Khan I.A., Schwartzman J.D., Kasper L.H., Moretto M. (1999). CD8+ CTLs are essential for protective immunity against *Encephalitozoon cuniculi* infection. J. Immunol..

[47] Rodríguez-Tovar L.E., Castillo-Velázquez U., Arce-Mendoza A.Y., Nevárez-Garza A.M., Zarate-Ramos J.J., Hernández-Vidal G., Rodríguez-Ramírez H.G., Trejo-Chávez A. (2016). Interferon γ and interleukin 10 responses in immunocompetent and immunosuppressed New Zealand White rabbits naturally infected with *Encephalitozoon cuniculi*. Dev. Comp. Immunol..

[48] Khan I.A., Moretto M. (1999). Role of gamma interferon in cellular immune response against murine *Encephalitozoon cuniculi* infection. Infect. Immun..

[49] Didier E., Shadduck J. (1994). IFN-gamma and LPS induce murine macrophages to kill *Encephalitozoon cuniculi* in vitro. J. Eukaryot. Microbiol..

[50] Duangurai T., Reamtong O., Thiangtrongjit T., Jala S., Chienwichai P., Thengchaisri N. (2025). Serum proteomic changes in pet rabbits with subclinical and clinical encephalitozoonosis in Thailand. Animals.

[51] Desoubeaux G., Piqueras M.D.C., Pantin A., Bhattacharya S.K., Peschke R., Joachim A., Cray C. (2017). Application of mass spectrometry to elucidate the pathophysiology of *Encephalitozoon cuniculi* infection in rabbits. PLoS ONE.

[52] Harcourt-Brown F.M., Holloway H.K. (2003). *Encephalitozoon cuniculi* in pet rabbits. Vet. Rec..

[53] Boot R., Hansen A.K., Hansen C.K., Nozari N., Thuis H.C. (2000). Comparison of assays for antibodies to *Encephalitozoon cuniculi* in rabbits. Lab. Anim..

[54] Cray C., Liebl M.P., Arheart K., Peschke R., Künzel F., Joachim A. (2020). Comparison of enzyme-linked immunosorbent assay and immunofluorescence test for determination of anti-*Encephalitozoon cuniculi* antibodies in sera from rabbits with different clinical and histopathological presentations. J. Exot. Pet Med..

[55] Desoubeaux G., Pantin A., Peschke R., Joachim A., Cray C. (2017). Application of Western blot analysis for the diagnosis of *Encephalitozoon cuniculi* infection in rabbits: Example of a quantitative approach. Parasitol. Res..

[56] Csokai J., Joachim A., Gruber A., Tichy A., Pakozdy A., Kunzel F. (2009). Diagnostic markers for encephalitozoonosis in pet rabbits. Vet. Parasitol..

[57] Cray C., McKenny S., Perritt E., Arheart K.L. (2015). Utility of IgM titers with IgG and C-reactive protein quantitation in the diagnosis of suspected *Encephalitozoon cuniculi* infection in rabbits. J. Exot. Pet Med..

[58] Cray C., Arcia G., Schneider R., Kelleher S.A., Arheart K.L. (2009). Evaluation of the usefulness of an ELISA and protein electrophoresis in the diagnosis of *Encephalitozoon cuniculi* infection in rabbits. Am. J. Vet. Res..

[59] Wicher V., Baughn R., Fuentealba C., Shadduck J., Abbruscato F., Wicher K. (1991). Enteric infection with an obligate intracellular parasite, *Encephalitozoon cuniculi*, in an experimental model. Infect. Immun..

[60] Waller T., Morein B., Fabiansson E. (1978). Humoral immune response to infection with *Encephalitozoon cuniculi* in rabbits. Lab. Anim..

[61] Owen D.G., Gannon J. (1980). Investigation into the transplacental transmission of *Encephalitozoon cuniculi* in rabbits. Lab. Anim..

[62] Vargas-Villavicencio J.A., Cañedo-Solares I., Correa D. (2022). Anti-*Toxoplasma gondii* IgM long persistence: What are the underlying mechanisms?. Microorganisms.

[63] Lennox A., Asahi Y., Arheart K., Ichiyanagi T., Cray C. (2020). Preliminary evaluation of an immunoturbidimetric assay and lateral flow device for the measurement of serum amyloid A in rabbits. J. Exot. Pet Med..

[64] Cray C., Rodriguez M., Fernandez Y. (2013). Acute phase protein levels in rabbits with suspected *Encephalitozoon cuniculi* infection. J. Exot. Pet Med..

[65] Lyngset A. (1980). A survey of serum antibodies to *Encephalitozoon cuniculi* in breeding rabbits and their young. Lab. Anim. Sci..

[66] Wright J.H., Craighead E.M. (1922). Infectious motor paralysis in young rabbits. J. Exp. Med..

[67] Škrbec M., Dovč A., Hrženjak N.M., Slavec B., Žlabravec Z., Kočar N., Rojs O.Z., Račnik J. (2023). *Encephalitozoon cuniculi* infection of domestic rabbits (*Oryctolagus cuniculus*) in Slovenia between 2017 and 2021. Pathogens.

[68] Valencakova A., Balent P., Petrovova E., Novotny F., Luptakova L. (2008). Encephalitozoonosis in household pet Nederland Dwarf rabbits (*Oryctolagus cuniculus*). Vet. Parasitol..

[69] Wang Y., Qin X., Diao X., Liu Y., Liu J. (2022). Serological survey for antibodies to *Encephalitozoon cuniculi* and *Toxoplasma gondii* in pet rabbits in eastern coastal areas of China. J. Vet. Med. Sci..

[70] Baldotto S.B., Cray C., Giannico A.T., Reifur L., Montiani-Ferreira F. (2015). Seroprevalence of *Encephalitozoon cuniculi* infection in pet rabbits in Brazil. J. Exot. Pet Med..

[71] Didier E.S., Didier P.J., Snowden K.F., Shadduck J.A. (2000). Microsporidiosis in mammals. Microb. Infect..

[72] Cox J.C., Gallichio H.A. (1978). Serological and histological studies on adult rabbits with recent, naturally acquired encephalitozoonosis. Res. Vet. Sci..

[73] Morsy E.A., Salem H.M., Khattab M.S., Hamza D.A., Abuowarda M.M. (2020). *Encephalitozoon cuniculi* infection in farmed rabbits in Egypt. Acta Vet. Scand..

[74] Rodríguez-Tovar L.E., Villarreal-Marroquín A., Nevárez-Garza A.M., Castillo-Velázquez U., Rodríguez-Ramírez H.G., Navarro-Soto M.C., Zárate-Ramos J.J., Hernández-Vidal G., Trejo-Chávez A. (2017). Histochemical study of *Encephalitozoon cuniculi* spores in the kidneys of naturally infected New Zealand rabbits. J. Vet. Diagn. Investig..

[75] Addie D.D., Tasker S., Boucraut-Baralon C., Belák S., Egberink H., Frymus T., Hartmann K., Hofmann-Lehmann R., Marsilio F., Lloret A. (2020). *Encephalitozoon cuniculi* infection in cats: European guidelines from the ABCD on prevention and management. J. Feline Med. Surg..

[76] Eidson M., Matthews S.D., Willsey A.L., Cherry B., Rudd R.J., Trimarchi C.V. (2005). Rabies virus infection in a pet guinea pig and seven pet rabbits. J. Am. Vet. Med. Assoc..

[77] Müller K., Fuchs W., Heblinski N., Teifke J.P., Brunnberg L., Gruber A.D., Klopfleisch R. (2009). Encephalitis in a rabbit caused by human herpesvirus-1. J. Am. Vet. Med. Assoc..

[78] Kazacos K.R., Reed W.M., Kazacos E.A., Thacker H.L. (1983). Fatal cerebrospinal disease caused by *Baylisascaris procyonis* in domestic rabbits. J. Am. Vet. Med. Assoc..

[79] Fisher P.G., Quesenberry K., Orcutt C., Mans C., Carpenter J.W. (2021). Neurologic and muscoskeletal diseases. Ferrets, Rabbits, and Rodents: Clinical Medicine and Surgery.

[80] Bertram A.C., Bertram B., Bartel A., Ewringmann A., Fragoso-Garcia A.M., Erickson A.N., Müller K., Klopfleisch R. (2021). Neoplasia and tumor-like lesions in pet rabbits (*Oryctolagus cuniculus*): A retrospective analysis of cases between 1995 and 2019. Vet. Pathol..

[81] Liatis T., Makri N., Czopowicz M., Richardson J., Nuttall T., Suñol A. (2024). Otitis media/interna and encephalitozoonosis are the most common causes of head tilt in pet rabbits in the UK: 73 cases (2009–2020). Vet. Rec..

[82] Doboși A.-A., Bel L.-V., Paștiu A.I., Pusta D.L. (2022). A review of *Encephalitozoon cuniculi* in domestic rabbits (*Oryctolagus cuniculus*)—Biology, clinical signs, diagnostic techniques, treatment, and prevention. Pathogens.

[83] Gruber A., Pakozdy A., Weissenbock H., Csokai J., Kunzel F. (2009). A retrospective study of neurological disease in 118 rabbits. J. Comp. Pathol..

[84] Mäkitaipale J., Järvenpää E., Bruce A., Sankari S., Virtala A.-M., Näreaho A. (2022). Seroprevalence of *Encephalitozoon cuniculi* and *Toxoplasma gondii* antibodies and risk-factor assessment for *Encephalitozoon cuniculi* seroprevalence in Finnish pet rabbits (*Oryctolagus cuniculus*). Acta Vet. Scand..

[85] Zanet S., Palese V., Trisciuoglio A., Alonso C.C., Ferroglio E. (2013). *Encephalitozoon cuniculi, Toxoplasma gondii* and *Neospora caninum* infection in invasive Eastern Cottontail Rabbits *Sylvilagus floridanus* in Northwestern Italy. Vet. Parasitol..

[86] Neumayerová H., Juránková J., Jeklová E., Kudláčková H., Faldyna M., Kovařčík K., Jánová E., Koudela B. (2014). Seroprevalence of *Toxoplasma gondii* and *Encephalitozoon cuniculi* in rabbits from different farming systems. Vet. Parasitol..

[87] Warnefors E., Rueløkke M., Gredal H. (2019). Results of a modified neurological examination in 26 healthy rabbits. J. Exot. Pet Med..

[88] Künzel F., Fisher P.G. (2018). Clinical signs, diagnosis, and treatment of *Encephalitozoon cuniculi* infection in rabbits. Vet. Clin. North Am. Exot. Anim. Pract..

[89] Eatwell K., Mancinelli E., Hedley J., Keeble E., Kovalik M., Yool D. (2013). Partial ear canal ablation and lateral bulla osteotomy in rabbits. J. Small Anim. Pract..

[90] Thomas W.B. (2000). Vestibular dysfunction. Vet. Clin. North Am. Small Anim. Pract..

[91] King A., Posthumus J., Hammond G., Sullivan M. (2012). Comparison of ultrasonography, radiography and a single computed tomography slice for the identification of fluid within the tympanic bulla of rabbit cadavers. Vet. J..

[92] Coeuriot C.T., Guise L., Cazin C.C., Meregalli R., Fusellier M.S. (2022). Tympanic bullae ultrasonography is feasible in nonsedated healthy rabbits (*Oryctolagus cuniculus*). J. Am. Vet. Med. Assoc..

[93] Richardson J., Longo M., Liuti T., Eatwell K. (2019). Computed tomographic grading of middle ear disease in domestic rabbits (*Oryctolagus cuniculi*). Vet. Rec..

[94] Rowley H.A., Uht R.M., Kazacos K.R., Sakanari J., Wheaton W.V., Barkovich A.J., Bollen A.W. (2000). Radiologic-pathologic findings in raccoon roundworm (*Baylisascaris procyonis*) encephalitis. Am. J. Neuroradiol..

[95] Isaac I., Richardson J., Liuti T., Longo M. (2022). Safety of intravenous iodinated contrast medium injection in rabbits undergoing conscious computed tomography. Vet. Rec..

[96] Abu-Akkada S.S., Oda S.S. (2016). Prevention and treatment of *Encephalitozoon cuniculi* infection in immunosuppressed rabbits with fenbendazole. Iran. J. Vet. Res..

[97] Suter C., Müler-Doblies U., Deplazes P., Hatt J.M. (2001). Prevention and treatment of *Encephalitozoon cuniculi* infection in rabbits with fenbendazole. Vet. Rec..

[98] Cray C., Altman N.H. (2022). An update on the biologic effects of fenbendazole. Comp. Med..

[99] Fisher P.G., Graham J.E., Carpenter J.W., Harms C. (2023). Rabbits. Carpenter’s Exotic Animal Formulary.

[100] Capece B., Alves M., Cristofol C., Hajovska K. (2009). Comparative embriotoxicity of albendazole and fenbendazole in rabbits. J. Vet. Pharmacol. Ther..

[101] Hedley J. (2020). BSAVA Small Animal Formulary; Part B: Exotic Pets.

[102] Burton M., Conway R., Mishkin N., Mama K., Knych H., Kendall L., Sadar M.J. (2023). Pharmacokinetics of gabapentin after single, oral administration in domestic rabbits (*Oryctolagus cuniculus*). J. Exot. Pet Med..

[103] Graham J.E., Garner M.M., Reavill D.R. (2014). Benzimidazole toxicosis in rabbits: 13 cases (2003 to 2011). J. Exot. Pet Med..

[104] Delk K.W., Carpenter J.W., KuKanich B., Nietfeld J.C., Kohles M. (2014). Pharmacokinetics of meloxicam administered orally to rabbits (*Oryctolagus cuniculus*) for 29 days. Am. J. Vet. Res..

[105] Jeklova E., Leva L., Jaglic Z., Faldyna M. (2008). Dexamethasone-induced immunosuppression: A rabbit model. Vet. Immunol. Immunopathol..

[106] Lee C., Jones T.A. (2021). Effects of several therapeutic agents on mammalian vestibular function: Meclizine, diazepam, and JNJ7777120. J. Assn. Res. Otolaryn..

[107] Eid R. (2018). Therapeutic review. J. Exot. Pet Med..

[108] Conway R.E., Desmarchelier M., Burton M., Mama K., Rao S., Kendall L.V., Sadar M.J. (2025). Single oral dose of gabapentin reduces vigilance and increases play behavior without changing mobility in New Zealand white rabbits (*Oryctolagus cuniculus*). J. Am. Vet. Med. Assoc..

[109] Ozawa S.M., Hawkins M.G., Drazenovich T.L., Kass P.H., Knych H.K. (2019). Pharmacokinetics of maropitant citrate in New Zealand White rabbits (*Oryctolagus cuniculus*). Am. J. Vet. Res..

[110] van Zeeland Y.R., Schoemaker N.J., Graham J.E., Doss G.A., Beaufrere H. (2021). Nutrition and fluid therapy. Exotic Animal Emergency and Critical Care Medicine.

[111] Harcourt-Brown F. (2002). Textbook of Rabbit Medicine.

[112] Matoba A.Y., McCulley J.P. (1985). The effect of therapeutic soft contact lenses on antibiotic delivery to the cornea. Ophthalmology.

[113] Frantz J.M., Dupuy B.M., Kaufman H.E., Beuerman R.W. (1989). The effect of collagen shields on epithelial wound healing in rabbits. Am. J. Ophthalmol..

[114] Eshar D., Wyre N., Schoster J. (2011). Use of collagen shields for treatment of chronic bilateral corneal ulcers in a pet rabbit. J. Small Anim. Pract..

[115] Chae J.J., Shin Y.J., Lee J.D., Seo K., Elisseeff J.H. (2018). Nictitating membrane fixation improves stability of the contact lens on the animal corneal surface. PLoS ONE.

[116] Xie H., Preast V. (2013). Xie’s Veterinary Acupuncture.

[117] Alessandrini M., Napolitano B., Micarelli A., De Padova A., Bruno E. (2012). P6 acupressure effectiveness on acute vertiginous patients: A double blind randomized study. J. Altern. Complement. Med..

[118] Margolin T. (2018). Resolution of vestibular disease in a lagomorph through acupuncture and herbs: A case report. J. Am. Holist. Vet. Med. Assoc..

[119] Schoen A. (2014). Integration of Acupuncture and Integrative Medicine Protocols for a More Comprehensive Approach to Veterinary Medicine. https://www.cabidigitallibrary.org/doi/pdf/10.5555/20143185068.

[120] Park S.-R., Han Y., Lee S.J., Lee K.-I. (2023). Efficacy of low-level laser therapy in a rabbit model of rhinosinusitis. Int. J. Mol. Sci..

[121] Millis D.L., Bergh A. (2023). A systematic literature review of complementary and alternative veterinary medicine: Laser therapy. Animals.

[122] Percy D.H., Barthold S.W. (2013). Pathology of Laboratory Rodents and Rabbits.

[123] Harcourt-Brown F.M. (2013). Diagnosis of renal disease in rabbits. Vet. Clin. Exot. Anim. Pract..

[124] Oliver-Guimera A., Asin J., Imai D.M., Casanova M.I., Strunk A., Keel K., Uzal F.A., Reavill D.R. (2024). Diseases of domestic rabbits by purpose; a retrospective study of 2,583 cases received at 4 diagnostic laboratories in California, USA, 2013–2022. J. Vet. Diagn. Investig..

[125] Erwingmann A., Göbel T. (1999). Examinations on clinics and therapy of encephalitozoonosis in pet rabbits. Kleintierpraxis.

[126] Brewer N.R., Cruise L.J., Manning P.J., Ringler D.H., Newcomer C.E. (1994). Physiology. The Biology of the Laboratory Rabbit.

[127] Cizek L.J. (1961). Relationship between food and water ingestion in the rabbit. Am. J. Physiol..

[128] Kozma C.K., Macklin W., Cummins L.M., Weisbroth S.H., Flatt R.E., Krause A.L. (1974). Anatomy, physiology, and biochemistry of the rabbit. The Biology of the Laboratory Rabbit.

[129] McLaughlin R.M., Fish R.E., Manning P.J., Ringler D.H., Newcomer C.E. (1994). Clinical biochemistry and hematology. The Biology of the Laboratory Rabbit.

[130] Jenkins J.R. (2010). Evaluation of the rabbit urinary tract. J. Exot. Pet Med..

[131] Keeble E., Benato L. (2013). Urinary tract surgery. BSAVA Manual of Rabbit Surgery, Dentistry and Imaging.

[132] Eken E., Çorumluoğlu Ö., Paksoy Y., Beşoluk K., Kalaycı İ. (2009). A study on evaluation of 3D virtual rabbit kidney models by multidetector computed tomography images. Anatomy.

[133] Hristov H., Kostov D., Vladova D. (2006). Topographical anatomy of some abdominal organs in rabbits. Trakia J. Sci..

[134] Sata Y., Burke S.L., Gueguen C., Lim K., Watson A.M., Jha J.C., Eikelis N., Jackson K.L., Lambert G.W., Denton K.M. (2020). Contribution of the renal nerves to hypertension in a rabbit model of chronic kidney disease. Hypertension.

[135] Gallego M. (2017). Laboratory reference intervals for systolic blood pressure, rectal temperature, haematology, biochemistry and venous blood gas and electrolytes in healthy pet rabbits. Open Vet. J..

[136] Strong-Townsend M., Fabian N., Skinner G., Murphy R., Hegarty E., Peterson S., Coyne M. (2024). Assay validation and determination of the reference interval for symmetric dimethylarginine in healthy rabbits. J. Exot. Pet Med..

[137] Özkan Ö., Yücesan B., Pekkaya S., Alçığır M.E., Gürcan İ.S. (2019). Relationship between seropositivity of *Encephalitozoon cuniculi* and renal biochemical markers in clinically healthy rabbits. Ank. Üniversitesi Vet. Fakültesi Derg..

[138] Sze-Yu Y., Chi-Hsuan S., Pin-Chen L., Ching-Fen W., Tsai-Lu L., Tsung-Li C., Chou C.-C. (2025). Urinary chemistry in healthy cross-bred pet rabbits (*Oryctolagus cuniculus*) and rabbits with suspected chronic kidney disease. Transl. Anim. Sci..

[139] Harcourt-Brown F. (2007). Radiographic signs of renal disease in rabbits. Vet. Rec..

[140] Banzato T., Bellini L., Contiero B., Selleri P., Zotti A. (2015). Abdominal ultrasound features and reference values in 21 healthy rabbits. Vet. Rec..

[141] Vilalta L., Altuzarra R., Espada Y., Dominguez E., Novellas R., Martorell J. (2017). Description and comparison of excretory urography performed during radiography and computed tomography for evaluation of the urinary system in healthy New Zealand White rabbits (*Oryctolagus cuniculus*). Am. J. Vet. Res..

[142] Porzio P., Pharr J.W., Allen A.L. (2001). Excretory urography by intraosseous injection of contrast media in a rabbit model. Vet. Radiol. Ultrasound.

[143] Altuzarra R., Vilalta L., Martorell J., Novellas R., Espada Y. (2018). Description of digital fluoroscopic excretory urography in healthy New Zealand rabbits (*Oryctolagus cuniculus*). Vet. Rec..

[144] Lippi I., Perondi F., Petrini D., La Fortuna M.C., Luci G., Intorre L., Guidi G., Meucci V. (2019). Evaluation of glomerular filtration rate estimation by means of plasma clearance of iohexol in domestic rabbits (*Oryctolagus cuniculus*). Am. J. Vet. Res..

[145] Michigoshi Y., Yamagishi N., Satoh H., Kato M., Furuhama K. (2011). Using a single blood sample and inulin to estimate glomerular filtration rate in rabbits. J. Am. Assn. Lab. Anim. Sci..

[146] Buch D., Saldanha A., Muehlbauer E., de Oliveira W.J., Gil E.M.U., Froes T.R. (2022). Computed tomographic findings of the urinary tract in rabbits (*Oryctolagus cuniculus*). J. Exot. Pet Med..

[147] Luo Z., Liu Y., Tang Z., Liu J., Xu X., Li M., Dai Y. (2021). Quantitative evaluation of renal cortex perfusion using contrast-enhanced ultrasound imaging parameters in ischemia–reperfusion injury in rabbits. Ultrasound Med. Biol..

[148] Sparkes A.H., Caney S., Chalhoub S., Elliott J., Finch N., Gajanayake I., Langston C., Lefebvre H.P., White J., Quimby J. (2016). ISFM consensus guidelines on the diagnosis and management of feline chronic kidney disease. J. Feline Med. Surg..

[149] Capello V. (2006). Diagnostic and therapeutic techniques in exotic pet practice. J. Exot. Pet Med..

[150] Di Girolamo N., Selleri P., Quesenberry K., Orcutt C.J., Mans C., Carpenter J.W. (2020). Disorders of the urinary and reproductive systems. Ferrets, Rabbits and Rodents: Clinical Medicine and Surgery.

[151] Smith M.V. (2013). Textbook of Rabbit Medicine.

[152] Henik R.A., Snyder P.S., Volk L.M. (1997). Treatment of systemic hypertension in cats with amlodipine besylate. J. Am. Anim. Hosp. Assoc..

[153] Flecknell P. (2009). Laboratory Animal Anaesthesia.

[154] Turner P.V., Chen C.H., Taylor M.W. (2006). Pharmacokinetics of meloxicam in rabbits after single and repeat oral dosing. Comp. Med..

[155] Fredholm D.V., Carpenter J.W., KuKanich B., Kohles M. (2013). Pharmacokinetics of meloxicam in rabbits after oral administration of single and multiple doses. Am. J. Vet. Res..

[156] Cowgill L.D., James K.M., Levy J.K., Browne J.K., Miller A., Lobingier R.T., Egrie J.C. (1998). Use of recombinant human erythropoietin for management of anemia in dogs and cats with renal failure. J. Am. Vet. Med. Assoc..

[157] Zhang J.F., Wu Y.L., Xu J.Y., Ye W., Zhang Y., Weng H., Shi W.D., Xu G.X., Lu L., Dai W. (2008). Pharmacokinetic and toxicity study of intravitreal erythropoietin in rabbits. Acta Pharmacol. Sin..

[158] Gallego M., Cassez N., Blanco A., Desmarchelier M., Benito J. (2019). Owner-assessed indices of quality of life (QOL) in rabbits and the relationship to the presence of painful disease: Exploratory study. Odontology.

[159] Tschudin A., Clauss M., Codron D., Liesegang A., Hatt J.M. (2011). Water intake in domestic rabbits (*Oryctolagus cuniculus*) from open dishes and nipple drinkers under different water and feeding regimes. J. Anim. Physiol. Anim. Nutr..

[160] An P., Dang H.-M., Shi X.-M., Ye B.-Y., Wu X.-L. (2014). “Qufeng Tongluo” acupuncture prevents the progression of glomerulonephritis by decreasing renal sympathetic nerve activity. J. Ethnopharmacol..

[161] Paterno J.C., Freire A.O., Soares M.F., Franco M.F., Schor N., Teixeira V.P.C. (2009). Electroacupuncture and moxibustion attenuate the progression of renal disease in 5/6 nephrectomized rats. Kidney Blood Press. Res..

[162] Koh R. How to use acupuncture and herbal medicine for treatment of chronic renal failure. Proceedings of the World Small Animal Veterinary Association Congress.

[163] Zhang Z.L., Ji X.Q., Zhang P., Zhang X.H., Meng Z.J., Yang X.J. (2007). Randomized and controlled study on needling method of harmonizing spleen-stomach for early intervention of diabetic nephropathies and the mechanism of protecting kidney. Zhongguo Zhen Jiu.

[164] Yu J.-S., Ho C.-H., Wang H.-Y., Chen Y.-H., Hsieh C.-L. (2017). Acupuncture on renal function in patients with chronic kidney disease: A single-blinded, randomized, preliminary controlled study. J. Altern. Complement. Med..

[165] Huang H., Zhang J., Gui F., Liu S., Zhong C., Wang T., Du H., He X., Cao L. (2021). Development of a simple single-acupoint electroacupuncture frame and evaluation of the acupuncture effect in rabbits. Vet. Sci..

[166] An P., Sun W.-S., Wu X.-L., Shi X.-M., Wang Z. (2012). Effect of acupuncture on renal function and pathologic changes of kidney in rabbits with nephritis. Zhongguo Zhen Jiu= Chin. Acupunct. Moxibustion.

[167] Liu Z.-H., Xu Q.-Y., Wang Y., Gao H.-X., Min Y.-H., Jiang X.-W., Yu W.-H. (2024). Catalpol from *Rehmannia glutinosa* targets Nrf2/NF-κB signaling pathway to improve renal anemia and fibrosis. Am. J. Chin. Med..

[168] Qiu H., Fan W., Fu P., Zuo C., Feng P., Liu F., Zhou L., Chen F., Zhong H., Liang Y. (2013). General acteoside of Rehmanniae leaves in the treatment of primary chronic glomerulonephritis: A randomized controlled trial. Afr. J. Tradit. Complement. Altern. Med..

[169] Zaaba N.E., Al-Salam S., Beegam S., Elzaki O., Yasin J., Nemmar A. (2023). Catalpol attenuates oxidative stress and inflammation via mechanisms involving sirtuin-1 activation and NF-κB inhibition in experimentally-induced chronic kidney disease. Nutrients.

[170] Lee H.S., Kim S.T., Cho D.K. (1993). Effects of rehmanniae radix water extract on renal function and renin secretion rate in unanesthetized rabbits. Am. J. Chin. Med..

[171] Yu S., Paetau-Robinson I. (2006). Dietary supplements of vitamins E and C and β-carotene reduce oxidative stress in cats with renal insufficiency. Vet. Res. Commun..

[172] Hu J., Liu Z., Zhang H. (2017). Omega-3 fatty acid supplementation as an adjunctive therapy in the treatment of chronic kidney disease: A meta-analysis. Clinics.

[173] Ingram A.J., Parbtani A., Clark W.F., Spanner E., Huff M.W., Philbrick D.J., Holub B.J. (1995). Effects of flaxseed and flax oil diets in a rat-5/6 renal ablation model. Am. J. Kidney Dis..

[174] Sener G., Paskaloglu K., Satiroglu H., Alican I., Kaçmaz A., Sakarcan A. (2004). L-carnitine ameliorates oxidative damage due to chronic renal failure in rats. J. Cardiovasc. Pharmacol..

[175] Ahmad N.A., Armaly Z., Berman S., Jabour A., Aga-Mizrachi S., Mosenego-Ornan E., Avital A. (2016). l-Carnitine improves cognitive and renal functions in a rat model of chronic kidney disease. Physiol. Behav..

[176] George S.M., Yassa H.A., Hussein H.A., El Refaiy A.M. (2017). Protective effect of L-carnitine against formaldehyde-induced kidney, liver and testicular damage in rabbits, a histopathological study. Mansoura J. Foren. Med. Clin. Toxicol..

[177] HM E., Citil M., Tuzcu M., Atakisi E., Gunes V., Uzlu E. (2009). The effect of L-carnitine administration on doxorubicine induced hepatoxicity and nephrotoxicity in rabbits. Kafkas Üniversitesi Veteriner Fakültesi Dergisi.

[178] Keller J., Ringseis R., Priebe S., Guthke R., Kluge H., Eder K. (2011). Dietary L-carnitine alters gene expression in skeletal muscle of piglets. Mol. Nutr. Food Res..

[179] Dipineto L., Rinaldi L., Santaniello A., Sensale M., Cuomo A., Calabria M., Menna L.F., Fioretti A. (2008). Serological survey for antibodies to *Encephalitozoon cuniculi* in pet rabbits in Italy. Zoonoses Public Health.

[180] Lavazza A., Chiari M., Nassuato C., Giardiello D., Tittarelli C., Grilli G. (2016). Serological investigation on *Encephalitozoon cuniculi* in pet rabbits in North-Central Italy. J. Exot. Pet Med..

[181] Hofmann-Wellenhof S., Nell B. (2024). Retrospective study on *Encephalitozoon cuniculi* infections in 118 cat and 9 dog eyes. Vet. Ophthalmol..

[182] Lin J., Nell B., Horikawa T., Zarfoss M. (2022). Feline intralenticular *Encephalitozoon cuniculi*: Three cases from California. J. Feline Med. Surg..

[183] Didier E.S., Weiss L.M. (2011). Microsporidiosis: Not just in AIDS patients. Curr. Opin. Infect. Dis..

[184] Shadduck J.A., Bendele R., Robinson G.T. (1978). Isolation of the causative organism of canine encephalitozoonosis. Vet. Pathol..

[185] Benz P., Maass G., Csokai J., Fuchs-Baumgartinger A., Schwendenwein I., Tichy A., Nell B. (2011). Detection of *Encephalitozoon cuniculi* in the feline cataractous lens. Vet. Ophthalmol..

[186] Keeble E., Shaw D. (2006). Seroprevalence of antibodies to *Encephalitozoon cuniculi* in domestic rabbits in the United Kingdom. Vet. Rec..

[187] Künzel F., Joachim A. (2010). Encephalitozoonosis in rabbits. Parasitol. Res..

[188] Giordano C., Weigt A., Vercelli A., Rondena M., Grilli G., Giudice C. (2005). Immunohistochemical identification of *Encephalitozoon cuniculi* in phacoclastic uveitis in four rabbits. Vet. Ophthalmol..

[189] Yuschenkoff D., Graham J., Pumphrey S.A. (2020). Diagnosis and treatment of glaucoma in client-owned rabbits (*Oryctolagus cuniculus*): 16 eyes from 11 rabbits (2008–2019). J. Exot. Pet Med..

[190] Sandmeyer L.S., Bauer B.S., Grahn B.H. (2011). Diagnostic ophthalmology. Can. Vet. J..

[191] Nell B., Csokai J., Fuchs-Baumgartinger A., Maaß G. (2015). *Encephalitozoon cuniculi* causes focal anterior cataract and uveitis in dogs. Tierärztliche Praxis Ausgabe K Kleintiere/Heimtiere.

[192] Felchle L.M., Sigler R.L. (2002). Phacoemulsification for the management of *Encephalitozoon cuniculi*-induced phacoclastic uveitis in a rabbit. Vet. Ophthalmol..

[193] Wolfer J., Grahn B., Wilcock B., Percy D. (1993). Phacoclastic uveitis in the rabbit. Prog. Vet. Comp. Ophthalmol..

[194] Stiles J., Didier E., Ritchie B., Greenacre C., Willis M., Martin C. (1997). *Encephalitozoon cuniculi* in the lens of a rabbit with phacoclastic uveitis: Confirmation and treatment. Vet. Comp. Ophthalmol..

[195] Baney V., Blacklock B., Keeble E. (2021). A review of ocular *Encephalitozoon cuniculi* infection in the rabbit. Comp. Anim..

[196] Sanchez R.F., Everson R., Hedley J., Dawson C., Lam R., Priestnall S.L., Garcia de Carellan A., de Miguel C., Seymour C. (2018). Rabbits with naturally occurring cataracts referred for phacoemulsification and intraocular lens implantation: A preliminary study of 12 cases. Vet. Ophthalmol..

[197] Gomes F.E., de Matos R., Ledbetter E. (2018). Phacoemulsification of bilateral cataracts in two pet rabbits. Open Vet. J..

[198] Goulle F., Bodin P., Cousin H., Prieto M., Cassagnes C. (2024). Bilateral phacoemulsification and intraocular lens implantation in a pet rabbit with cataracts. Open Vet. J..

[199] Gwon A., Tsonis P.A. (2008). The rabbit in cataract/IOL surgery. Animal Models in Eye Research.

[200] Garcia-Sanchez P., Romero-Trancón D., Sainz T., Calvo C., Iglesias I., Perez-Hernando B., Hurtado-Gallego J., Sánchez R., Alcolea S., Moya L. (2024). The role of veterinarians in zoonosis prevention: Advising families of immunocompromised children with pets. One Health.

[201] Garcia-Sanchez P., Aguilar-Valero E., Sainz T., Calvo C., Iglesias I., Bueno D., Frauca E., Ramos-Boluda E., Alcolea-Sanchez A., García-Guereta L. (2023). Immunocompromised children and young patients living with pets: Gaps in knowledge to avoid zoonosis. Transbound. Emerg. Dis..

[202] Abou-El-Naga I., Gaafar M., Gomaa M.M., El Achy S.N. (2020). *Encephalitozoon intestinalis*: A new target for auranofin in a mice model. Med. Mycol..

[203] Guruceaga X., Perez-Cuesta U., Abad-Diaz de Cerio A., Gonzalez O., Alonso R.M., Hernando F.L., Ramirez-Garcia A., Rementeria A. (2019). Fumagillin, a mycotoxin of *Aspergillus fumigatus*: Biosynthesis, biological activities, detection, and applications. Toxins.

[204] Wei J., Fei Z., Pan G., Weiss L.M., Zhou Z. (2022). Current therapy and therapeutic targets for microsporidiosis. Front. Microbiol..

[205] Pinheiro A., Neves F., Lemos de Matos A., Abrantes J., van der Loo W., Mage R., Esteves P.J. (2016). An overview of the lagomorph immune system and its genetic diversity. Immunogenetics.

[206] Oppelt C., Starkloff A., Rausch P., Von Holst D., Roedel H.G. (2010). Major histocompatibility complex variation and age-specific endoparasite load in subadult European rabbits. Mol. Ecol..

[207] Aseeja P., Shaikh Y., Bajpai A., Sirsikar P., Kalra S.K. (2021). Advancement in our understanding of immune response against *Encephalitozoon* infection. Parasite Immunol..

